# Optic-nerve-transmitted eyeshine, a new type of light emission from fish eyes

**DOI:** 10.1186/s12983-017-0198-9

**Published:** 2017-02-27

**Authors:** Roland Fritsch, Jeremy F. P. Ullmann, Pierre-Paul Bitton, Shaun P. Collin, Nico K. Michiels

**Affiliations:** 1 Institute of Evolution and Ecology, University of Tübingen, 72076 Tübingen, Baden-Württemberg Germany; 2 Centre for Advanced Imaging, University of Queensland, Brisbane, 4072 Queensland Australia; 3 Department of Neurology, Boston Children’s Hospital & Harvard Medical School, Boston, MA 02115 USA; 4 School of Biological Sciences and the Oceans Institute, University of Western Australia, Crawley, 6009 Western Australia Australia

**Keywords:** Marine visual ecology, Eye anatomy, Eyeshine, Optic nerve, Light guidance, Tripterygiidae, *Tripterygion delaisi*

## Abstract

**Background:**

Most animal eyes feature an opaque pigmented eyecup to assure that light can enter from one direction only. We challenge this dogma by describing a previously unknown form of eyeshine resulting from light that enters the eye through the top of the head and optic nerve, eventually emanating through the pupil as a narrow beam: the Optic-Nerve-Transmitted (ONT) eyeshine. We characterize ONT eyeshine in the triplefin blenny *Tripterygion delaisi* (Tripterygiidae) in comparison to three other teleost species, using behavioural and anatomical observations, spectrophotometry, histology, and magnetic resonance imaging. The study’s aim is to identify the factors that determine ONT eyeshine occurrence and intensity, and whether these are specifically adapted for that purpose.

**Results:**

ONT eyeshine intensity benefits from locally reduced head pigmentation, a thin skull, the gap between eyes and forebrain, the potential light-guiding properties of the optic nerve, and, most importantly, a short distance between the head surface and the optic nerves.

**Conclusions:**

The generality of these factors and the lack of specifically adapted features implies that ONT eyeshine is widespread among small fish species. Nevertheless, its intensity varies considerably, depending on the specific combination and varying expression of common anatomical features. We discuss whether ONT eyeshine might affect visual performance, and speculate about possible functions such as predator detection, camouflage, and intraspecific communication.

**Electronic supplementary material:**

The online version of this article (doi:10.1186/s12983-017-0198-9) contains supplementary material, which is available to authorized users.

## Background

**Fig. 1:**
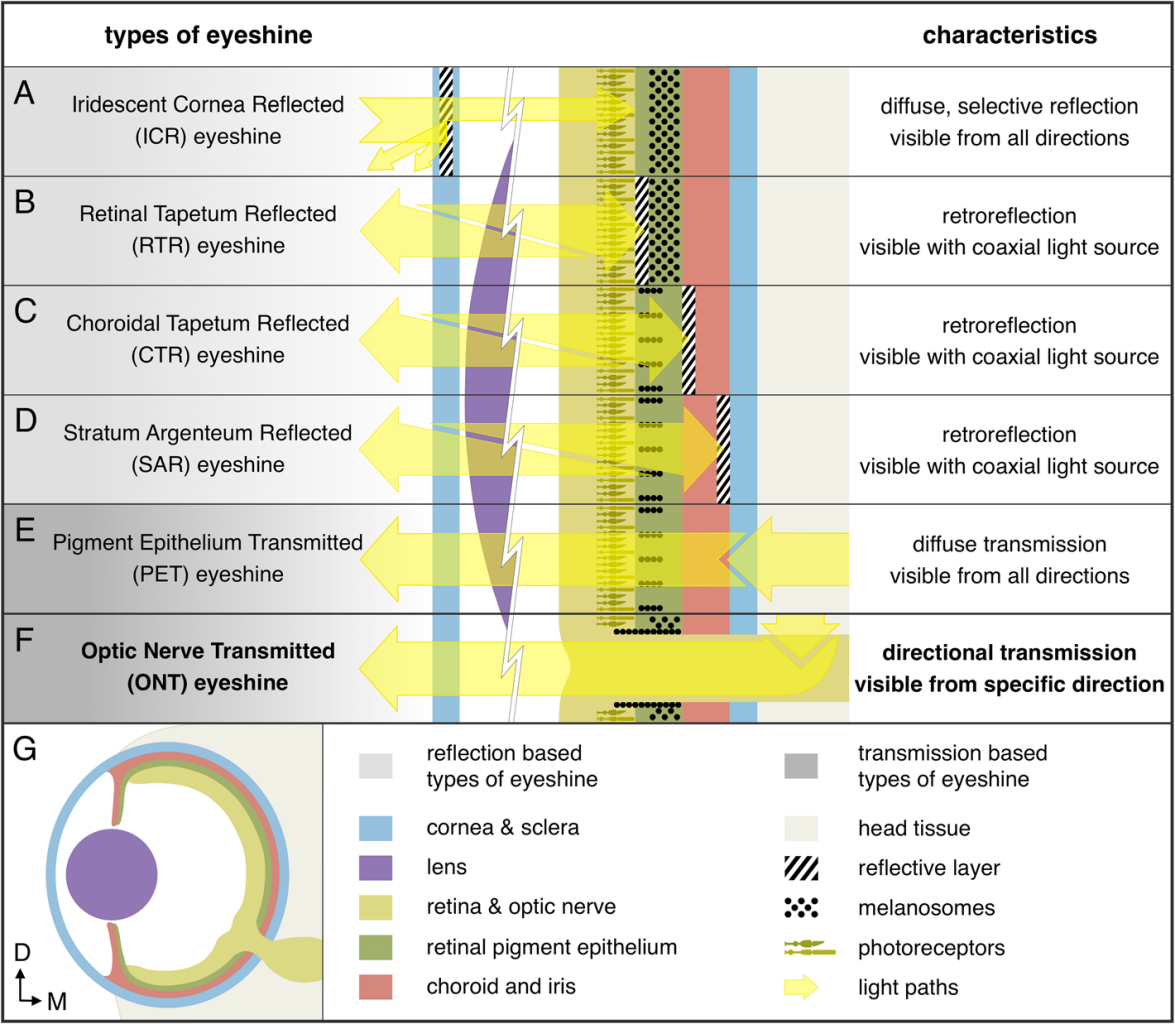
Types of eyeshine in vertebrate eyes. **a** ICR eyeshine constitutes a case of iridescence produced by arrays of regularly arranged collagen fibres in the cornea. Light reflection is only partial, specular, and both wavelength- and angle-dependent. **b** Retinal-tapetum-reflected (RTR) eyeshine is caused by an intracellular *tapetum lucidum* in the inner part of the RPE. Tapetal reflection can be specular and/or diffuse. The eyes’ optics project the reflected light back to its source, an effect known as retroreflection. **c** In CTR eyeshine, the *tapetum lucidum* lies within the inner portion of the choroid. For light to reach the tapetum, the RPE must transmit light at least partially (e.g., through melanosome motility). The choroidal tapetum can be cellular (specialised, reflective cells) or fibrous (regularly arranged, extracellular collagen fibres), both of which can generate strong retroreflection. **d** SAR eyeshine has similar properties and requirements as CTR, but is caused by the *stratum argenteum*, a distinct, cellular, reflective layer in the outer choroid. **e** PET eyeshine occurs when the RPE and choroid are (partially) unpigmented, and the eyes are in an exposed, dorsal position. Ambient light shines through the fundus and is seen as PET eyeshine from a wide range of directions. PET eyeshine regularly co-occurs with SAR eyeshine. **f** In optic-nerve-transmitted (ONT) eyeshine, light enters the eye through the ON. Pigmentation of the RPE and choroid are irrelevant for ONT eyeshine, but pigment sheaths around the intraocular ON protect the photoreceptors from transmitted light. ONT eyeshine can co-occur with any other type of eyeshine. Since the light is emitted from the optic disc, its projection and visibility are restricted to a narrow beam of the same shape as the disc. **g** Cross-section through an eye for a general overview of the involved structures. *D* dorsal, *M* medial

Vision implies the presence of photoreceptors that absorb and transform light energy into a neural signal that can be interpreted by the brain. Advanced visual abilities as in vertebrates, however, also depend on the presence of the melanin containing retinal pigment epithelium (RPE) behind the photoreceptors. It absorbs excess light to prevent scattering within the eye and shields the photoreceptors against light coming from behind the eye, improving image contrast and resolution. This explains why the pupils of camera-type eyes are typically black. Some eyes, however, do not function strictly unidirectionally and may show stunningly bright pupils, from which light appears to be emitted by the eye, a phenomenon called *eyeshine*. Figure 1 provides an overview of previously described types of eyeshine [1, 2] and the new type described in this study. Based on the general mechanism, we broadly categorize the different types as either reflection-based (Fig. 1a-d), or transmission-based (Fig. 1e and f).

Reflection-based eyeshine can either be caused by an iridescent cornea (Fig. 1a), or a reflective layer within the eye (Fig. 1b-d) [1]. Iridescent-cornea-reflected (ICR) eyeshine differs from the other types in that it is produced outside the eyecup. It occurs in many shallow-water fishes and is assumed to serve flare reduction, as filter, or to camouflage the pupil [3]. Reflective layers behind the photoreceptors cause the well-known eyeshine featured in many animals, cats being a prominent example [1, 2]. There is variation in the layer type involved (*tapetum lucidum*, *stratum argenteum*), the location of the reflectors (RPE, inner choroid, outer choroid), what reflective structures are responsible (extracellular fibres or intracellular platelets, needles, cuboids, spherules), and how these reflect the incident light (diffusely or specularly) [1, 2, 4]. In all cases the eye seems to glow from within because light, which is not absorbed by the retinal photoreceptors, is reflected back through the photoreceptors. The remaining light is then emitted out of the pupillary aperture along the incident optic pathway. In eyes with focusing optics, the reflection is directed towards the light’s original source, an effect called retroreflection. Consequently, the reflection is visible only to an observer whose viewing axis is near-coaxial with the illuminating light [5]. These forms of reflective eyeshine (types B-D, Fig. 1) increase the retina’s probability of photon capture by doubling the effective length of the photoreceptors’ outer segments. This is particularly useful under dim light for crepuscular, nocturnal, and deep sea species [1]. Alternatively, reflection-based eyeshine might also allow species to maintain a certain photon capture efficiency while reducing investment in receptor length and photopigment density [6]. Other possible functions include improvement of polarization sensitivity [7] and camouflage in the pelagic environment [8].

Choroidal-tapetum-reflected (CTR; Fig. 1c) and stratum-argenteum-reflected eyeshine (SAR; Fig. 1d) require the RPE to be at least partially translucent. In scorpionfishes (Scorpaenidae) and toadfishes (Batrachoididae) this is achieved by the melanosomes congregating in RPE cell processes enveloping the cones, leaving the somata transparent, and thus allowing incoming light to reach the *argenteum* [9]. If, additionally, the eye bulges out of the skull dorsally and exposes its back, downwelling light can partially pass through, causing pigment-epithelium-transmitted (PET) eyeshine (Fig. 1e). To an observer, the resultant eyeshine is similar to reflective eyeshine, but without the retroreflective property. The effect is generally less bright due to light absorption while passing through several layers of tissue. Its occurrence in the toadfish’s light-adapted eyes questions photon-catch enhancement and favours camouflage as probable function [9].

Here, we characterize a previously undescribed phenomenon of apparent light emission from the eyes as a new type of eyeshine. It is generated by ambient light that passes through the dorsal cranium and the extra- and intraocular sections of the optic nerve (ON) into the eye. There it is emitted from the optic disc, the area where all ganglion cell axons converge to form the ON, and leaves the eye through the pupil. Due to this light path, we call it optic-nerve-transmitted (ONT) eyeshine (Fig. 1f). Even though ONT eyeshine is also transmission-based, its properties differ crucially, and it can occur independently of the presence of the other types, even when RPE and choroid are densely pigmented, as the ON bypasses both. ONT eyeshine does not involve reflection and thus does not require illumination from the direction of the observer. Because the transmitted light is projected into the environment by the lens, the effect can only be seen from a specific, narrow angle. We describe ONT eyeshine qualitatively and quantitatively in the triplefin *Tripterygion delaisi*, a species with particularly strong ONT eyeshine. To determine whether *T. delaisi* evolved special features that facilitate its ONT eyeshine, we examine the anatomical structures along the light path using spectrophotometry, histology, and magnetic resonance imaging (MRI). Specifically, we investigated whether general skull structure increases transmittance, whether the ON’s morphological characteristics could allow it to act as a light guide, and whether the optic disc shows features that prevent or control interference of the ONT eyeshine with vision. To ascertain whether these structures and characteristics are specific adaptations only found in *T. delaisi*, or just common features of small fishes, we compared its cranial and ocular anatomy with that of three similarly sized teleost species with varying combinations of traits that might influence ONT eyeshine: *Tripterygion melanurus* (Tripterygiidae) is closely related and very similar to *T. delaisi* but has a more densely pigmented head; *Parablennius zvonimiri* (Blenniidae) is more distantly related but shares a similar, crypto-benthic ecology; the black-and-white morph of *Amphiprion ocellaris* (Pomacentridae) was included as an assumed negative control. Of the studied species, it is most distantly related to *T. delaisi*, belongs to a family of free-swimming, bentho-pelagic fishes, and was also chosen for having the darkest head pigmentation among the study species. These few species cannot and are not meant to provide a comprehensive analysis, but should suffice to reveal the most relevant factors that generate and modify the ONT eyeshine.

Finally, we discuss whether the phenomenon is simply a by-product of small size or if it could serve a function, like detection of targets featuring reflective eyeshine, camouflage, intraspecific communication, or enhancing visual performance.

## Results and discussion

Due to the diversity of techniques used in this study and of factors involved in ONT eyeshine, we combine the presentation and discussion of the gathered data by the main relevant aspects. Data are presented as mean ± standard deviation. In-text we refer to the summarized data sets in Tables 1, 2 and 3. A comprehensive overview of all anatomical data can be found in the additional online material.

**Table 1:** Summarized absolute and relative anatomical data of specimens used for histology and MRI (see also Figs. 5 and 6)

Species	Value type	Total length [mm]	Head diameter [mm)	Body volume [mm^3^]	Skull (bone) thickness [μm] (% of HD)	Dermis thickness [μm] (% of HD)	ON depth [mm] (% of HD)	ON layers [N]	ON layer thickness [μm] (% of ONØ]	ON CSA [mm^2^] (% of head CSA)
*T. delaisi*	absolute	53.6 ± 4.6	6.9 ± 0.4	684 ± 133	54.0 ± 6.9	94.2 ± 8.0	1.57 ± 0.04	7.8 ± 0.8	54.9 ± 13.4	0.135 ± 0.040
	relative				(0.76 ± 0.09)	(1.33 ± 0.12)	(22.1 ± 2.0)		(13.2 ± 2.1)	(0.35 ± 0.07)
10	*N*	10	10	10	3	3	3	3	3	3
*T. melanurus*	absolute	40.5 ± 5.1	4.9 ± 06	258 ± 86	38.3 ± 7.5	35.3 ± 6.2	1.09 ± 0.05	6.5 ± 0.7	40.1 ± 11.1	0.065 ± 0.035
	relative				(0.78 ± 0.01)	(0.72 ± 0.004)	(22.3 ± 3.0)		(14.2 ± 0.03)	(0.36 ± 0.12)
4	*N*	4	4	4	2	2	2	2	2	2
*P. zvonimiri*	absolute	42	5.55	339	84.2 ± 54.7^a^	260.6 ± 160.4^a^	1.38 ± 0.21^a^	4.5^a^	63.5 ± 8.4^a^	0.124 ± 0.037^a^
	relative				(1.52 ± 0.99)^a^	(4.70 ± 2.89)^a^	(24.9 ± 3.8)^a^		(16.0 ± 2.1)^a^	(0.51 ± 0.15)^a^
1	*N*	1	1	1	1	1	1	1	1	1
*A. ocellaris*	absolute	52.9 ± 5.2	10.0 ± 1.2	1419 ± 503	255.5 ± 39.5	275.0 ± 80.9	2.16 ± 0.31	10.2 ± 0.8	42.7 ± 4.3	0.096 ± 0.007
	relative				(2.50 ± 0.21)	(2.67 ± 0.52)	(21.1 ± 1.6)		(12.2 ± 1.7)	(0.12 ± 0.03)
9	*N*	9	9	9	4	4	4	3	3	3

Data represent mean ± SD of N individuals or of repeated measurements within single individual (^a^). Reduced N caused by splitting individuals over different methods. *CSA* cross-sectional area, head CSA calculated as $\frac{\pi}{4}$ *HD* ^2^; *HD* head diameter, calculated as mean of head width and height; *ON* optic nerve; *ON* Ø optic nerve diameter

**Table 2:** Summarized spectrophotometric measurements data of ONT eyeshine transmission efficiency (see also Fig. 3)

Species (*N*)	Standard length [mm]	Head diameter [mm]	ON depth [mm]	DWS [photons/s/sr/mm^2^]	ONT eyeshine [photons/s/sr/mm^2^] (relative to DWS)	PET eyeshine [photons/s/sr/mm^2^] (relative to DWS)
*T. delaisi* (5)	46.4 ± 3.8	7.3 ± 0.9	1.61 ± 0.15	1.51 ± 0.34 ×10^19^	3.15 ± 1.81 ×10^17^ (0.0203 ± 0.0088)	7.01 ± 2.23 ×10^16^ (0.0047 ± 0.0010)
*T. melanurus* (2)	30.5 ± 6.4	4.4 ± 0.6	0.98 ± 0.13	1.04 ± 0.11 ×10^19^	2.09 ± 0.46 ×10^17^ (0.0205 ± 0.0065)	2.24 ± 0.55 ×10^16^ (0.0021 ± 0.0003)
*P. zvonimiri* (5)	38.6 ± 6.5	6.0 ± 1.2	1.49 ± 0.23	1.47 ± 0.17 ×10^19^	1.56 ± 0.12 ×10^17^ (0.0108 ± 0.0018)	3.04 ± 1.12 ×10^16^ (0.0022 ± 0.0011)
*A. ocellaris* (5)	25.5 ± 2.2	5.6 ± 0.1	1.18 ± 0.09	1.46 ± 0.16 ×10^19^	1.66 ± 0.63 ×10^17^ (0.0112 ± 0.0036)	1.13 ± 0.26 ×10^16^ (0.0008 ± 0.0001)

ON depth was estimated by applying relative ON depths from Table 1 to head diameters in this data set. Head diameter was calculated as the mean of head width and height measurements. *DWS* diffuse white standard, *sr* steradian

**Table 3:** Optic nerve torsion data summarised per genus, compare Fig. 6

Genus	Number	ON length [mm]	Starting angle [°]	Torsion [°/mm]	Total torsion [°]
*Tripterygion*	5	0.81 ± 0.16	19.3 ± 9.6	93.4 ± 8.2	75.5 ± 20.5
*Parablennius*	1	0.96	12.5	78.7	75.7
*Amphiprion*	3	1.40 ± 0.09	11.6 ± 20.0	82.5 ± 12.2	116.2 ± 24.8

Parameters were first averaged per individual (not shown), then per genus. *T. delaisi* and *T. melanurus* were grouped because of their virtually identical internal anatomy

### General mechanism and properties of ONT eyeshine

**Fig. 2:**
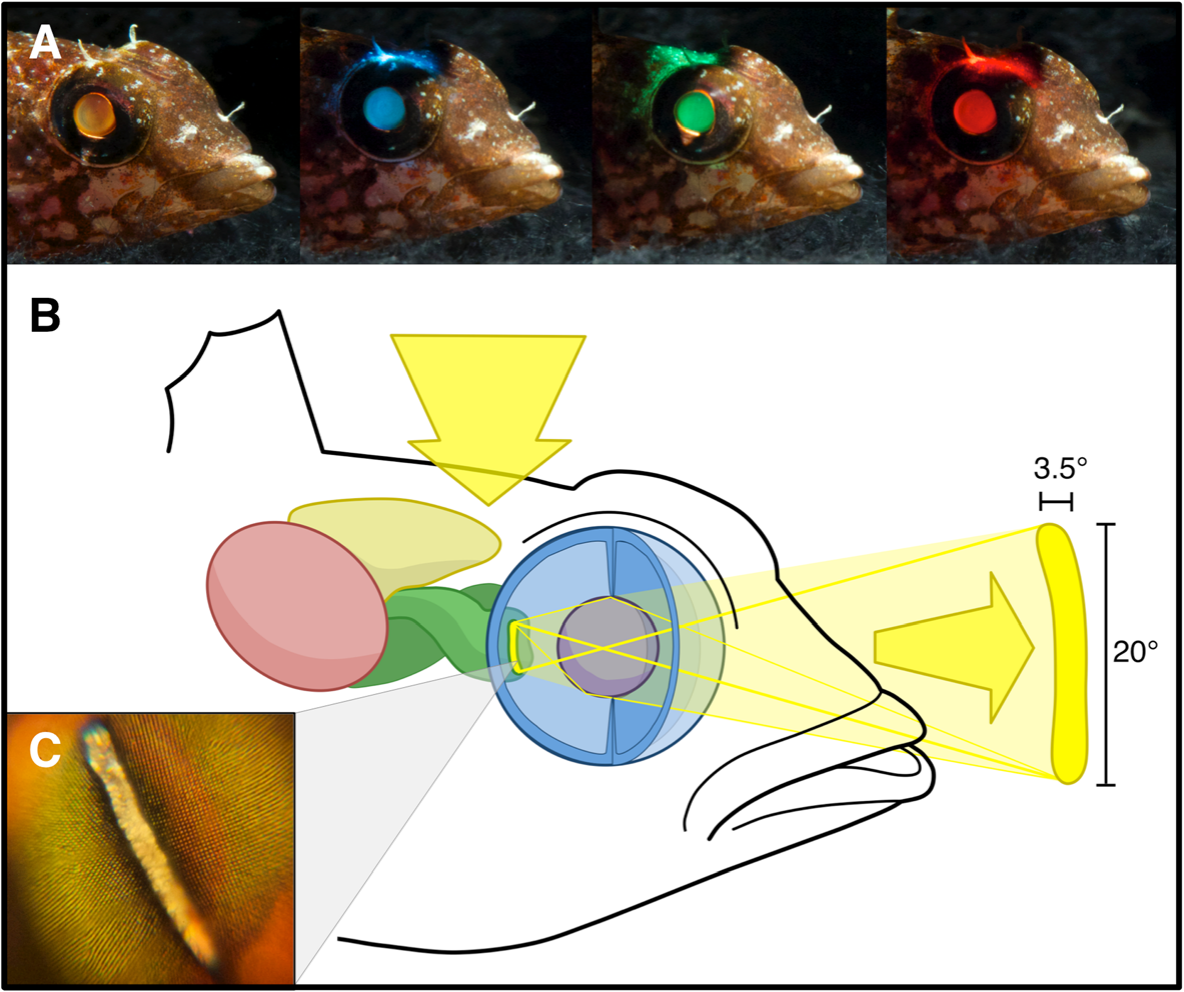
Laboratory demonstration and origin of ONT eyeshine. **a** *Tripterygion delaisi* illuminated dorsally with different spectra, illustrating that the ONT eyeshine is generated by skull illumination and based on transmission of the incident light (Additional file 2). **b** Schematic light path of ONT eyeshine in *T. delaisi*. Light, which enters through the dorsal head surface, passes through the anterior brain and extraocular ONs, exits through the optic disc, and is projected into the environment as a narrow beam of light. *red*: optic tectum; *buff*: telencephalon; *green*: optic nerves; *blue*: eye; *purple*: lens; *yellow*: ONT eyeshine light path. **c** View into the eye of *T. delaisi* through an endoscope (without internal light source) under the same illumination as in A_white_, showing the light-emitting, elongated optic disc (see also Additional file 3). A similar image can be seen directly with the human eye. Without small-aperture or micro-lens optics (e.g., endoscopes), the entire pupil seems to emit light since the optic disc’s image is blurred as a consequence of focusing on the outside of the fish with a wide aperture lens (as in a, taken with DSLR camera)

**Fig. 3:**
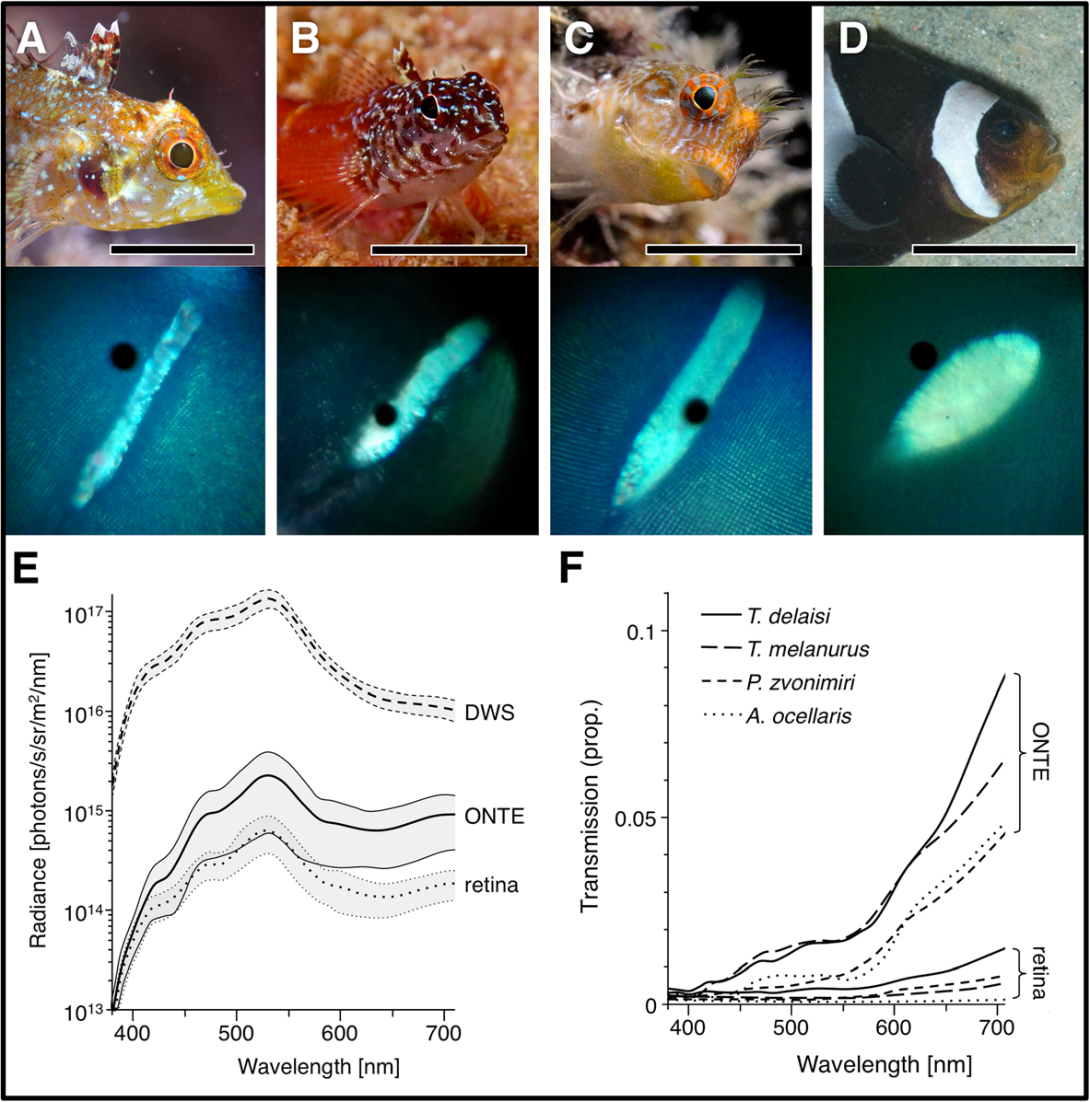
Studied species and their ONT eyeshine. **a**-**d** Habitus and optic disc (OD) shape of *Tripterygion delaisi* (**a**), *Tripterygion melanurus* (**b**), *Parablennius zvonimiri* (**c**), and *Amphiprion ocellaris* (**d**). Fish images a, b, and c were taken in the field, d in the laboratory. *Scale bars* equal 1 cm. OD images were taken through a spectrophotometer with attached endoscope under dorsal illumination of the fish’s head. The *black dot* is the detection area of the spectrophotometer and was positioned within the OD during measurements. The bluish tint is due to the cyan filter used for illumination. **e** Absolute radiance measurements (*n* = 5, mean ± SD) of a diffuse white reflectance standard (DWS), *T. delaisi*’s optic disc with ONT eyeshine (ONTE), and the adjacent retinal PET eyeshine, all under the same conditions (see Additional file 6). The DWS served as a proxy for illumination intensity. **f** Mean transmission efficiency of the study species’ ONT eyeshine and retinal background relative to the DWS (*T. melanurus*: *n* = 2, all others: *n* = 5). Transmission is poor for short-wavelength light in all species, but consistently rises with increasing wavelength. *T. delaisi* features the highest proportional transmission, reaching about 8% at the red end of the visible spectrum

For ONT eyeshine to occur, a fish’s head must be subjected to downwelling light. A portion of that light is transmitted through the head and is in part collected by the ON, passes through the optic disc, and exits the eye through the lens and pupil (Fig. 2; Additional files 1 and 2). Inspecting the retina of *T. delaisi* and the other study species with an endoscope while illuminating the fish’s head dorsally demonstrates that the light indeed emanates from the optic disc (Fig. 3a-d; Additional file 3). Entering the eye through the ON and optic disc means that ONT eyeshine can be produced independently of a tapetum, stratum argenteum, or partially light-transmissive pigment epithelium.Additional file 1: Video of ONT eyeshine in *T. delaisi* in its natural environment. Footage of *T. delaisi* displaying ONT eyeshine under ambient light conditions in its natural habitat. The pupil is not completely dark because *T. delaisi’*s eyes also feature a certain degree of PET eyeshine. For the brief moment the ONT light shines directly at the camera, its much greater brightness and conspicuousness become obvious. Footage was taken in Corsica, 2012. (MP4 2899 kb)
Additional file 2: Video of ONT eyeshine in the laboratory under alternating illumination. A live *T. delaisi* was filmed while kept in a small observation tank, positioned so that the ONT eyeshine was oriented towards the camera. The fish was illuminated through two fibre optic cables connected to independent cold light sources. One provided blue, general illumination, while the other was specifically aimed at the top of the fish’s head, and its illumination colour was changed using the inbuilt filters of the cold light source. The video shows that the colour of the ONT eyeshine corresponds to the light illuminating the head surface, proving that the ONT light must indeed be transmitted through the head and nerve tissue. Compare Fig. 3a. (MOV 8824 kb)
Additional file 3: Video of *T. delaisi’s* optic disc viewed through an endoscope. The optic disc and surrounding retinal region of a live *T. delaisi* were filmed through the fish’s pupil using an endoscope attached to a Nikon D4 camera. The fish was only illuminated from above, and not through the endoscope. The greenish glow of the retina represents PET eyeshine, while the bright appearance of the optic disc is due to ONT light, which is externally perceived as ONT eyeshine. (MOV 1172 kb)

ONT eyeshine further differs in two crucial aspects from the other transmission-based (PET) eyeshine. First, it is emitted as a narrow beam in a specific direction, a consequence of the fact that it is focused by the lens, becoming a projection of the optic disc’s image. The resulting ONT eyeshine beam extended over 3.5 ± 0.3° (*n* = 6) horizontally and 19.6 ± 3.3° (*n* = 5) vertically in *T. delaisi*, mirroring the optic disc’s elongated shape (Figs. 2c and 3a). Second, ONT eyeshine is brighter than PET eyeshine. While PET light needs to pass the sclera, choroid, pigment epithelium, and retina, which in combination absorb or scatter most of the light, ONT light is transmitted through *T. delaisi*’s unpigmented intraocular ON and thus bypasses all above tissue layers. This was supported by the results of the spectrophotometric measurements of ONT and PET eyeshine (Table 2). *T. delaisi*’s optic disc transmitted on average 2.03 ± 0.88% (*n* = 5) of incident light (approximated as the radiance of a diffuse white standard in the same position), while the surrounding retina’s transmission was 0.47 ± 0.10%, i.e., PET eyeshine was only one fourth as bright as ONT eyeshine (Fig. 3e and f). *T. melanurus* had a very similar ONT eyeshine transmission (2.05 ± 0.65%, *n* = 2), but a much weaker PET eyeshine with only one tenth that transmission. Contrary to our expectations, both the individuals of the blenny *P. zvonimiri* and the clownfish *A. ocellaris* exhibited marked ONT eyeshine during the spectrophotometric measurements. *P. zvonimiri*’s ONT eyeshine was the dimmest, with 1.08 ± 0.18% (*n* = 5) transmission, and one fifth that much PET light passing through the surrounding retina. *A. ocellaris* featured a comparably bright ONT eyeshine (1.12 ± 0.36%, *n* = 5), but virtually no PET eyeshine as its retina transmitted only one fifteenth of that. The bright ONT eyeshine in *A. ocellaris*, our assumed negative control, can be attributed to the small size of the individuals of *A. ocellaris* available for measurement. The individuals used for anatomical investigations were twice the size and showed no visually discernible eyeshine (compare Tables 1 and 2). Specific transmission increased markedly with wavelength in all species (Fig. 3f), presumably due to the lower absorption and scattering of longer wavelength light in biological tissue [10].

### Light transmissive features of the skull

**Fig. 4:**
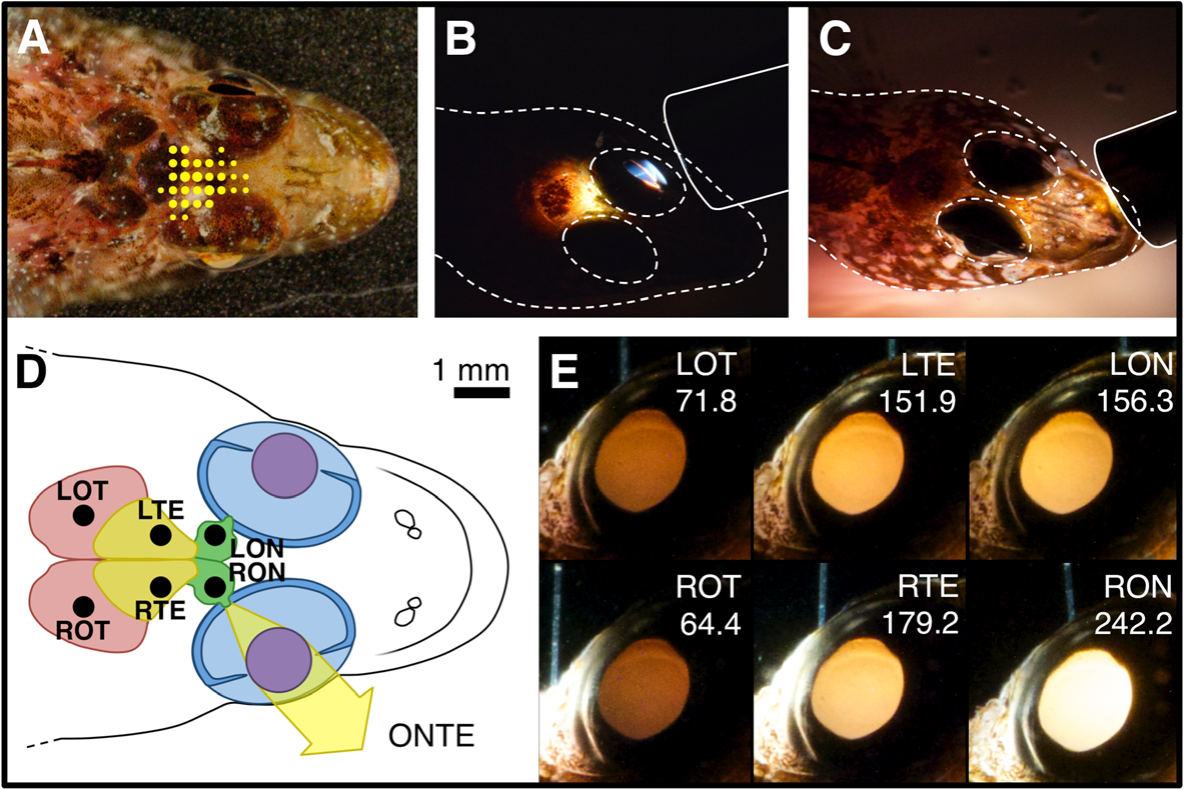
ONT eyeshine excitation efficiency and light path in *T. delaisi.* **a** Excitation map showing area where ONT eyeshine can be induced by dorsal illumination with small light spots (ca. 1 mm^2^). *Dot size* corresponds with perceived intensity of ONT eyeshine in the right eye. **b** Reversing the light path, i.e., shining light through the (*left*) pupil of a recently sacrificed fish, produces glow corresponding to the maximum excitation field just behind the eye (compare a). Position of glass fibre is shown as *solid line*, head contour as *dashed line*. **c** Aiming the glass fibre light at the snout makes most of the head glow, except for the eyes. **d** Schematic of *T. delaisi*’s head in dorsal view. The *black dots* represent the positions of ONT eyeshine excitation, the result of which is shown in **e**). *LOT*/*ROT* *left/right* optic tectum, *LTE*/*RTE* left/right half of telencephalon, *LON*/*RON* *left/right* optic nerve, *ONTE* optic-nerve-transmitted eyeshine. **e** ONT eyeshine intensity in the right eye of a recently sacrificed *T. delaisi* with dorsal cranium removed. The tip of a single glass fibre was placed over different brain areas, as specified in **d**). The images show the correspondent ONT eyeshine emerging in the right eye. *Numbers* indicate pupil mean grey value as a proxy for ONT eyeshine brightness. All images were taken with a DSLR camera and identical settings

**Fig. 5:**
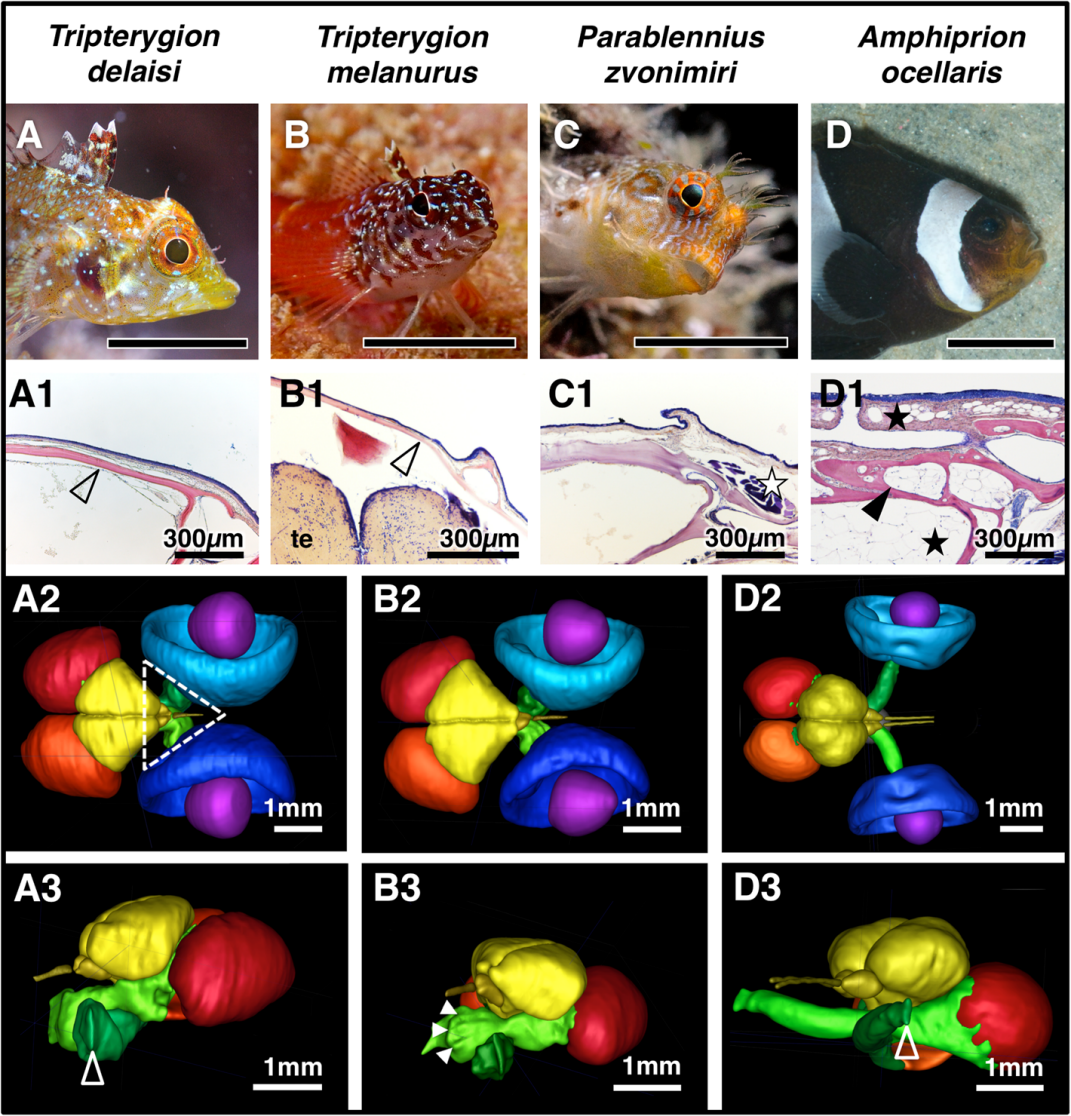
Skull structure and visual system of *T. delaisi* (**a**), *T. melanurus* (**b**), *P. zvonimiri* (**c**), and *A. ocellaris* (**d**; *scale bars* 1 cm). **a1**-**d1** Close-up of the dorsal skull and dermis. Triplefins have delicate but solid bones (**a1**-**b1**, *open arrowheads*; *te* telencephalon) compared to the thicker, chambered bones (**d1**, *black arrowhead*) of the clownfish. The skull is only covered by a thin dermis in triplefins, while the blenny has muscles running between the dermis and the skull (**c1**, *white star*), and the clownfish has fatty tissue (**d1**, *black stars*) throughout its head. **a2**-**d3** MRI segmentations of inner head anatomy in *T. delaisi*, *T. melanurus*, and *A. ocellaris:* retinae (*blue*), lenses (*purple*), ONs and tracts (*green*), optic tecta (*red*), telencephalon, olfactory nerves and bulbs, and diencephalon (*yellow*). *T. delaisi* (**a2**, **a3**) and *T. melanurus* (**b2**, **b3**) are practically identical, except for differences in absolute size. **a2**-**d2** Dorsal view. The *dashed triangle* frames the exposed part of the ON, which coincides with the area of most efficient ONT eyeshine induction (see Fig. 4a). In *A. ocellaris* (**d2**, **d3**), the telencephalon covers a smaller part of the nerves, but these lie deeper in the head. **a3**-**d3** Frontolateral view onto the left optic disc. While narrow and elongated in triplefins, it is shorter and elliptical in the clownfish (*open arrowheads*). The ridges along the nerve surface in **b3** (*solid arrowheads*) result from the triplefins’ loose ON pleats. The clownfish’s ON is smooth in comparison, due to the denser pleating

Systematic illumination of ventral and dorsal point locations of the head of *T. delaisi* identified a small, triangular region directly between and behind the eyes that produced the strongest ONT eyeshine (Fig. 4a). Four features of this region facilitate light transmission. First, the skin and cranial bones are extremely thin (Fig. 5a1; Table 1; 94.2 ± 8.0 μm and 54.0 ± 6.9 μm, *n* = 3). Corrected for head size, these values correspond to 1.33 ± 0.12% and 0.76 ± 0.09% of the head diameter, which is much thinner than those of *Parablennius zvonimiri* and *Amphiprion ocellaris* (Table 1). Second, the skin is only partially pigmented (Fig. 4a). Third, fat tissue, which would scatter light more strongly than other tissue because of its higher refractive index, is absent from the skin and skull (Fig. 5a1). Fourth, the interstitial gap between skull, eyes, and telencephalon leaves the anterior ON exposed to light entering the head (Fig. 5a2).

*T. melanurus* is morphologically very similar to *T. delaisi*, except for differences in colouration and size (Fig. 5b-b3). The equally strong ONT eyeshine (Table 2) could result from the used individuals’ smaller head size compensating the darker head pigmentation (Fig. 5b). The other species’ weaker ONT eyeshine can be explained by increased pigmentation, thicker skin, and thicker skull bones (Fig. 5c, c1, d, and d1). In addition, *A. ocellaris*’ bones are chambered and parts of its skull contain fat tissue (Fig. 5d1), both of which might increase light loss through scattering due to the heterogeneity of the tissue and its refractive index. The fact that *P. zvonimiri* and *A. ocellaris* still produce ONT eyeshine half as strong as that of the triplefins, suggests that head size is the dominant factor, followed by head structure. Pigmentation seems to be of secondary importance relative to these two. Ultimately, it is a constructive combination of all of these factors that produces the strongest ONT eyeshine, which seems to be the case in the two triplefins.

### Extraocular optic nerve pathway and light guidance

The optic nerve (ON) is formed by all ganglion cell axons, which converge at the optic disc and form the intraocular ON that crosses the retina, RPE, choroid, and scleral eyecup. Outside the eye it continues as the extraocular ON, whose features and how they relate to the ONT eyeshine are discussed in this section. The intraocular nerve will be discussed in the next section.

The strength of ONT eyeshine will decrease with increasing distance between head surface and ON (referred to as ON depth from here on), because a longer light path increases losses through scattering and absorption. The measured ON depths of the specimens used for histology varied from 1.09 ± 0.05 mm (*n* = 2) in *T. melanurus* to 2.16 ± 0.31 mm (*n* = 4) in *A. ocellaris* (Table 1). Expressed relative to mean head diameter, however, ON depth was similar across all four species (Table 1). This suggests that *T. delaisi*’s small ON depth is not specifically adapted to enhance ONT eyeshine, but results from the correlation with its small head and body size, both of which can be small for many other reasons [11]. Comparing the ONT eyeshine intensity with ON depths shows no obvious correlation (Table 2). Hence, although a small ON depth certainly contributes to strong ONT eyeshine, its effect can be modulated by e.g., pigmentation as in the case of *T. melanurus*.

Many biological tissues exhibit light guiding properties under certain conditions and to varying extents [12–18]. We argue that this also applies to the ON. Light guidance is usually assumed to arise from total internal reflection of light within structures that exhibit a difference in refractive index between internal and external medium, as was the case for the light guidance properties of the dentine layer in vertebrate teeth [19]. More recent studies, however, suggest that the underlying mechanism is repeated anisotropic scattering along the low-refractive-index tubules embedded in the high-refractive-index dentine tissue, and that this phenomenon may also occur in neural tissue [15, 20]. A confirmatory finding showed that Müller cells appear to funnel light arriving at the inner retina towards individual photoreceptors [12, 14]. Whatever the mechanism, transitions in refractive indices are a key prerequisite for light guiding. The ON’s structure creates such transitions on three different, yet interconnected, levels of anatomical detail.

**Fig. 6:**
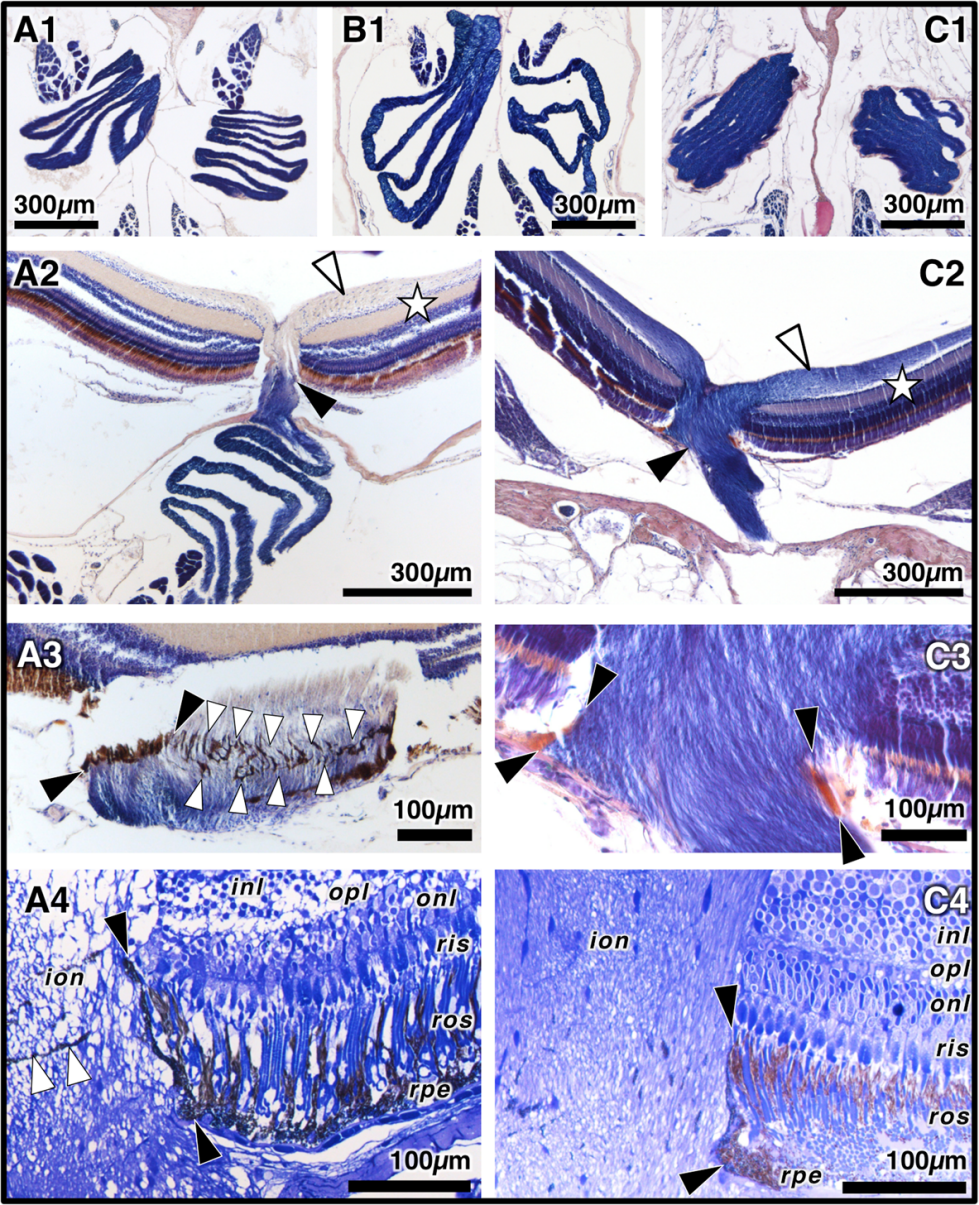
Optic nerve characteristics of *T. delaisi* (**a1**-**a4**), *P. zvonimiri* (**b1**), and *A. ocellaris* (**c1**-**c4**). **a1**-**c1** Transverse sections showing elaborately pleated ONs that differ in pleat number, thickness, regularity, and spacing. In *T. delaisi* (**a1**), pleats are further apart than in *A. ocellaris* (**c1**) and more evenly spaced than in *P. zvonimiri* (**b1**). This regular pattern of alternating high-refractive-index neural tissue and low-refractive-index interstitial spaces may enable the ON to act as a biological light guide (*T. melanurus' * ON structure is identical to *T. delaisi*'s). **a2**-**c2** Coronal sections of the intra- and extraocular ON in *T. delaisi* (a2) and *A. ocellaris* (c2). The PTAH staining, which stains myelin dark blue, confirms the common pattern that axons are unmyelinated within the nerve fibre layer (NFL) of the retina (*white arrowhead*) and become myelinated outside the eyecup (*black arrowhead*) in *T. delaisi*. The axons of *A. ocellaris* stained dark blue throughout both the intraocular ON and the NFL of the retina (*black* and *white arrowhead*), indicating that at least some of them are myelinated within the NFL. **a3**-**c3** Detail of the intraocular ON (coronal section). The RPE forms a pigment sheath (*black arrowheads*) that surrounds the ON in both *T. delaisi* (**a1**) and *A. ocellaris* (**b1**). In *T. delaisi*, the sheath comprises a distinct band of pigmented processes that criss-crosses the entire intraocular ON (*white arrowheads*). **a4**-**c4** Semi-thin sections of the intraocular ON confirm that the pigment sheath (*black arrowheads*) is an extension of the RPE in both species. The sheath extends just far enough to shield the light-sensitive photoreceptor outer segments (ros) (between the *black arrowheads*). Within-nerve pigment processes (*white arrowheads*) seem to be only present in *T. delaisi*. Abbreviations: *inl* inner nuclear layer, *ion* intraocular optic nerve, *onl* outer nuclear layer, *opl* outer plexiform layer, *ris* receptor inner segments, *ros* receptor outer segments, *rpe* retinal pigment epithelium

1. ONs contain parallel-running myelinated axons. Due to their high lipid and protein content, myelin sheaths have a high refractive index (*n* = 1.455) compared to neural cell somata (*n* = 1.358) and the aqueous extracellular medium (*n* ≅ 1.335) [14, 21]. Myelinated axons have been shown to trap and guide laser light through internal reflection [22], but they also constitute high-refractive-index tubular microstructures and thus fulfil the requirements for anisotropic scattering as described for dentine [15].

2. Nerves are interspersed with connective tissue, consisting of bundles and layers of collagen fibres. Their high refractive index, compared to the surrounding cytoplasm and extracellular matrix, and regular arrangement can make collagenous layers reflective enough to serve as *tapetum lucidum*, as they do in ungulates [4]. Thus, they might contribute to internal reflection in the ON as well.

3. Teleost ONs are usually ribbon-shaped and pleated [23], which creates alternating layers of nerve tissue and interstitial space, which constitutes yet another level of interfaces with changes in refractive index that might enhance internal reflection. We found this pleated-ribbon-like ON shape with 4–11 layers, each 30–60 μm thick, prominently present in all four study species (Fig. 6a1 to c1; Table 1). The ONs in *T. delaisi* and *T. melanurus* feature relatively widely and regularly spaced layers directly behind the eyes (Fig. 6a1). The ONs of *P. zvonimiri* feature few and well-separated layers, compared to *A. ocellaris*’s twice as many and more densely packed pleats (Table 1). The fact that their ONT eyeshine intensities are similar to each other, yet lower than those of the two triplefins (Table 2), could indicate that many layers with regular spacing are optimal for ONT eyeshine generation.

Additionally, the above features are non-exclusive in enabling and promoting light guidance. Combined they may act synergistically and generate a stronger effect than what the reflectivity of the individual structures would predict. An in-depth analysis and quantitative assessment of the extent of light guidance by the ON, however, is beyond the scope of this initial study, but should be addressed in future experiments.

Further factors that should impact the ON’s capacity to capture and guide light are its cross-sectional shape and orientation, especially if the pleated ribbon structure plays a major role. In *T. delaisi*, *T. melanurus*, and *P. zvonimiri*, the ON widens immediately behind the eye, exposing a larger area to incoming light (Fig. 5a2-3 and b2-3; Fig. 6b1). In contrast, the ONs of *A. ocellaris* have a round cross-section that does not change much in diameter upon leaving the eye (Fig. 5d2-3). The orientation of the ONs varied along their paths to the brain in all species examined (Table 3; Additional file 4). The rate of torsion (degrees per millimetre of ON) was similar when averaged at the genus level (78.2 to 93.4°/mm), but showed considerable variation between individuals, and even between the two nerves of the same individual. In the first third of the ON, immediately behind the eye, however, the pleats were close to horizontal in all species (Table 3). In a stack of horizontal layers, downwelling light would have to pass all reflective interfaces to make it through the nerve, thus maximising the chances and proportion of light to be reflected internally. With increasing tilt of the layers, however, downwelling light would pass fewer and fewer interfaces, thus reducing the chances of reflection. Additionally, the ON layers expose their maximal surface area to the downwelling light when they are horizontal. Given the torsion found in all study species, however, the orientation becomes suboptimal further down the ON’s path to the brain. The combination of a widened ON and nearly horizontal nerve layers directly behind the eye locally maximizes the potential to capture incoming light and further supports why ONT eyeshine can be induced most strongly in that specific area.Additional file 4: Video of *T. delaisi’s* optic nerve torsion. Video produced from the aligned and stacked images of transverse serial sections of *T. delaisi*’s and *A. ocellaris’* heads (see Methods section for more details). The clip shows the ON’s pleating, layer orientation, and trajectory from exiting the eyecup to past the optic chiasm, where the nerves become the optic tracts. The two species also differ in the surrounding tissue, especially thickness and structure of the skull. (MP4 7632 kb)


Two observations support the hypothesised directional propagation of light in *T. delaisi*’s ONs. First, when we reversed the light path by shining light into the pupil, only a limited area of the head lit up (Fig. 4b) that corresponded closely with the area of ONT eyeshine excitation (Fig. 4a). By comparison, pointing the same light source at the same fish’s snout made the whole head glow, demonstrating random scattering within non-neural tissue (Fig. 4c). Second, we illuminated specific areas of a dead specimen’s brain to determine their contribution to the strength of the ONT eyeshine emanating from the right eye (Fig. 4d). If light propagation in neural tissue were completely isotropic, i.e., homogeneous, the distance between light source and the right eye’s optic disc should determine ONT eyeshine intensity. If light is scattered anisotropically, or even guided by the neural tissue as we assume, the neural pathway should correspond with the light’s path to some extent and affect ONT eyeshine intensity accordingly. Since the ONs cross at the chiasm, the right ON is mostly covered by the tip of the right telencephalon, but projects to the left optic tectum (and vice versa for the left ON). Figure 4e shows the unedited images, all taken with the same, fixed camera settings, of the ONT eyeshine that results from illuminating the spots indicated in Fig. 4d. The numbers represent the average grey value of the pupil area as a rough but objective measure for brightness. The resulting ONT eyeshine was consistently brighter when the illuminated spot lay along the neural path of the right ON, compared to similarly distant other spots. Specifically, the left ON and right telencephalon sites had the same distance to the right optic disc, yet the ONT eyeshine from the right telencephalon was clearly brighter; the left telencephalon site was further away than the left ON site, yet both produced almost equally bright ONT eyeshine; the left optic tectum had a slightly greater distance but still produced brighter ONT eyeshine than the right optic tectum. All these observations support a non-random component in the transmission of light through the neural tissue and the optic nerve in particular.

In summary, it seems plausible that ONs guide light. Whether this is achieved through internal reflection, anisotropic scattering, or a combination of both, is as yet unclear. The structures that allow for the above mechanisms are common to the optic nerve architecture in most teleosts [23]. Bearing in mind that all investigated species produced measurable ONT eyeshine, the slight differences in their optic nerve structure cannot be regarded the main determinant of ONT eyeshine occurrence and intensity. Nevertheless, the relatively large light-exposed surface, as well as the regularly pleated, widely spaced, and horizontally oriented ON layers found in triplefins may increase the nerve’s ability to capture and guide light, thus posing an additional contribution to the triplefins’ strong ONT eyeshine.

### Intraocular optic nerve and photoreceptor shielding

The intraocular ON of all four species is surrounded by a pigmented sheath formed by the RPE who’s main functions are to prevent backscattering of light not absorbed by photoreceptors and to block light from reaching the photoreceptors through the fundus from the back of the eye. Apparently, that function extends to shielding the photosensitive outer segments of nearby photoreceptors from light leaking from the ON (Fig. 6a3 and c3). We found additional RPE processes within the intraocular ON in both *Tripterygion* species, but not in *A. ocellaris* (Fig. 6a4 and c4). This might be due to a greater need to shield the photoreceptors from the stronger ONT light in the two triplefins compared to *A. ocellaris*. For *P. zvonimiri* we lack the required semi-thin histological sections to assess this aspect.

The optic discs varied in shape from a tapered oval in *A. ocellaris* to a long and narrow strip in *T. delaisi* (Fig. 3a-d). Optic disk size increases with axon number and degree of myelination, being smaller with unmyelinated axons [24]. Being a blind spot, it seems reasonable to assume selection for a minimal size. This constraint and the retrograde myelination of fish ONs, during ontogeny [25] and regeneration [26], may explain why only the extraocular part of the axons is myelinated in most fishes. PTAH staining, which differentially dyes several tissue types and substances, including myelin in deep blue, confirmed this pattern in *T. delaisi*, *T. melanurus,* and *P. zvonimiri*, whose axons are unmyelinated in the intraocular ON and the axons were homogenously and densely packed (Fig. 6a2). From the perspective of light travelling through the ON towards the retina, both the axon’s myelin sheaths and the regular structure with layers of alternating tissue types gradually disappear along the intraocular ON. This removes the differences in refractive index needed for light guiding and presumably allows ONT light to eradiate from the optic disc area and leave the eye as ONT eyeshine rather than being guided towards the photoreceptors. In contrast, the retina of *A. ocellaris* possesses at least some intraocularly myelinated axons (Fig. 6c2), which is similar to previous findings in other vertebrate species [27–29]. When the axons’ myelin sheaths extend past the optic disc, light contamination may enter the retina. This could expose the photoreceptors to additional incident light and possibly affect the signal-to-noise ratio. Too much noise may impair perception (as discussed below). A lack of myelinated axons in the retina, as seen in *Tripterygion* and *Parablennius*, will reduce such noise interference.

### Possible functions of ONT eyeshine

**Fig. 7:**
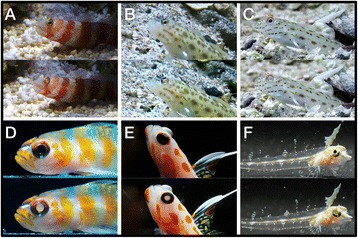
ONT eyeshine occurrence in other fish species. Additional examples of small, benthic fish species observed to exhibit ONT eyeshine: **a** *Amblyeleotris periophthalma*, **b** *Ctenogobiops pomastictus*, **c** *Ctenogobiops maculosus*, **d** *Trimma cana*, **e** *Stonogobiops yasha*, **f** *Enneapterygius pusillus. Upper* and *lower* images show the same individuals only moments apart. Slight differences in eye orientation direct the beam of ONT light either away from the observer and let the eyes appear normal (*upper* images), or towards the observer and reveal the ONT eyeshine (*lower* images). Pictures a-c were taken in the field, d-f in field station facilities; all courtesy of NKM

Thus far, we have merely described ONT eyeshine and the factors that determine its brightness. Our results suggest that the effect strongly depends on small body size and is modulated by head pigmentation, head anatomy, and ON structure. Given these simple requirements for the occurrence of ONT eyeshine, and that we did not find any unique adaptations that specifically allow or maximise ONT eyeshine, it follows that many sufficiently small and lightly pigmented species should exhibit and be affected by this phenomenon. Over the last few years of fieldwork and excursions, we indeed observed ONT eyeshine in more fishes than the four species that were investigated in detail here (Fig. 7), but such observations were sparse and capturing them on camera difficult. Several factors make it hard to spot ONT eyeshine in the field and may explain why it has been overlooked thus far: ONT light is emitted into the environment as a narrow beam in a forward direction. Benthic fishes usually avoid long eye contact, and most small fishes even turn away when approached. As a consequence, even if an observer spotted a fish at the right moment and from the perfect angle, the phenomenon is usually so ephemeral that it is likely dismissed as a passing reflection, if noticed at all. Therefore, it is understandable to question the significance of the effect and whether it is just a coincidental by-product that is inevitable in sufficiently small fishes. At this stage, we cannot exclude that option since none of *T. delaisi*’s anatomical features described in this study could be regarded as a specific adaptation solely evolved to enhance ONT eyeshine. Nevertheless, from the fish’s perspective, the effect is constantly present, and even a coincidental effect may have consequences and interact with other phenomena. Natural selection often exploits initially coincidental effects and recruits them for new functions, if they constitute a significant advantage to their bearers. Therefore, it is worthwhile not only to discuss the potential costs of ONT eyeshine’s interference with the visual system, but also to speculate about possible benefits and functions it might serve. We discuss four options we think warrant attention and may be tested in future studies.

#### Interactions with the visual system

It is intuitively tempting to assume ONT eyeshine affects visual performance negatively, as ONT light enters the eye from the seemingly wrong direction. If such light reached the photoreceptors, it would arrive scrambled and unfocussed since it never went through an optical system. Hence, it could certainly not serve image formation, but would nevertheless interact with other light and affect vision. Depending on the circumstances, the interference would not necessarily have to be detrimental. As a direct benefit, ONT light could still convey general information such as changes in ambient light caused by a passing shadow from a predator swimming by overhead. ONT light could also stimulate intrinsically photosensitive retinal neuronal cells that were found in some teleost retinas and might regulate circadian rhythms, modulate receptive fields, or tune the retinal circuitry to the dynamically changing aquatic light environment [30–32]. The possible contribution of ONT light can only be minor in this context, however, since the light reaching the retina through the conventional pathway stimulates the photosensitive cells as well. Teleosts also possess several photosensitive brain areas, not only in the pineal but also throughout the deep brain, that are involved in many regulatory processes [33]. Both the translucency of the skull and the light guidance by the optic nerves could originally be adaptations that allow light to reach those brain areas. ONT eyeshine may have originated as a side-effect of this, as there is no a priori reason to assume the ON would guide light in only one direction.

Another possible effect relates to the extreme flicker on benthic surfaces that is generated by wavelets on the surface on sunny days and which is a challenge for human eyes and digital cameras alike. That flicker results in synchronous fluctuations in the intensity of ONT eyeshine (personal observations; see also Additional file 1). Hence, ONT light may provide feedback to prime visual perception for illumination variance in a visual scene [34], improving vision under these conditions. Allowing ONT light to reach the rods and cones to achieve the above also means adding an unspecific, uncorrelated component to the total visual stimulus, i.e., increasing receptor noise levels. Therefore, the amount of ONT light reaching the receptors should be kept low and well regulated, which might be achieved by the unmyelinated axons inside the retina and by the RPE processes that extend into *T. delaisi*’ s intraocular ON. Otherwise, the aforementioned benefits would come at the cost of reduced general image contrast and acuity. Investigating these effects was beyond the scope of this study, but would be of interest for future research.

#### Predator and/or prey detection

Could ONT eyeshine be used as a searchlight? Reflective targets in the environment may reflect the emitted ONT eyeshine back to *T. delaisi*, which then might use this information for target detection. Such targets could be eyes that exhibit reflection-based eyeshine [1], which is indeed featured by both prey (e.g., small crustaceans, [35]) and predators (e.g., scorpion fishes, [1]) of *T. delaisi*. Reflective eyeshine can indeed be induced by weak light sources, as long as the distance between sender and target is short, as in small benthic fish searching for prey, and the light source is coaxial to the viewing axis, which applies almost perfectly to ONT eyeshine. It has previously been suggested that reflective eyeshine can be conspicuous and might alert potential predators [36], or enable private communication with conspecifics [37]. The practical use of ONT eyeshine in such a context has one intrinsic limitation. Since the light passes through the triplefin’s optical system both when emitted and returning, it is focused on the same retinal area where it originated, i.e., the optic disc, a blind spot. This restriction could be circumvented if the triplefin looked at a target with defocussed eyes, causing the target’s image to blur on the retina and excite some of the receptors adjacent to the optic disc. Consequently, alternating accommodation would make the target’s eye appear to blink.

#### Camouflage

Eyes are striking and attention-drawing structures, which is exemplified by the use of false eyes, i.e., eyespots, to deter or mislead predators and reduce predation, e.g., in butterflies [38] and fishes [39]. In cryptic animals, however, eyes can reduce camouflage [40] and some predators even specifically target eyes or eyespots [41]. Hence, the usual, round and black, shape of a pupil may be a conspicuous telltale sign for the presence of an otherwise cryptic species [8]. Similar to what has been suggested for corneal iridescence [3, 42], ONT eyeshine could reduce the contrast between iris and pupil by letting the latter appear bright and blend in with the luminance of the surrounding iris.

The narrow, beam-like shape of the ONT eyeshine, however, limits such a function. To achieve the assumed effect, *T. delaisi* would have to permanently point the ONT eyeshine, and thus its blind spot, at an observer and follow its movements, which could be done in principal, but with maximally two observers simultaneously, taking into account *T. delaisi*’s ability to move its eyes independently. Reflection-based and PET eyeshine may be more useful in a camouflage context, as they can be seen from much wider angles [1, 8, 43]. *T. delaisi* does also exhibit PET eyeshine, as demonstrated by the dimly green glowing retina when viewed through an endoscope (Figs. 2c and 3e-f). Both effects benefit from the translucent skull of *T. delaisi* and increase with ambient light levels and they are not mutually exclusive in their potential camouflage function.

#### Intraspecific communication

ONT eyeshine may also be a component of visual, intraspecific communication. To an observer, the ONT eyeshine does not differ from most other visual signals. Yet, two characteristics make it unique. First, sending a signal from the eye itself may generally pique an observer’s attention [40]. Second, emitting a narrow beam from the eye allows the sender to target a specific receiver. Minute eye movements in the sender will turn the signal on and off from the receiver’s perspective. To further assess this possible function, we modelled the perceived contrast between a pupil with and without ONT eyeshine as seen by another *T. delaisi*. While ONT eyeshine does not generate a perceivable chromatic contrast, there is a clearly visible achromatic contrast at both 5 and 20 m depth, with 4.85 ± 2.1 JNDs and 5.04 ± 2.2 JNDs, respectively (Additional file 5). ONT eyeshine could thus serve as an easily visible, yet non-interceptable, private signal between specific individuals. Furthermore, this scenario requires the light to travel the distance between sender and receiver only once, and hence the signal will be perceivable over greater distances compared to the two-way path needed for active photolocation.

Since the narrow spread of the beam works to the fish’s advantage in this context, and the generated contrast is strong, this might be the most likely of the three discussed functions for ONT eyeshine.

## Conclusions

Optic-nerve-transmitted eyeshine is a previously undescribed phenomenon that lets diurnal fishes’ pupils glow. It results from collection of ambient light by the ON in the post-orbital head area. The light is then transmitted into the eye and emitted through the pupil into the environment. This one-way, transmission-based light path distinguishes ONT eyeshine from the conventional, reflection-based types of eyeshine. ONT eyeshine is strong in the triplefin *T. delaisi* due to the additive effects of several traits, the most important being the short distance between ONs and the head surface. The ONs of teleosts generally feature specific structural characteristics that suggest the potential to partially guide light. Leakage of light from ONT eyeshine to the photoreceptors is prevented by an extended pigment sheath around the intraocular ON and by the typical, unmyelinated retinal axons. As a consequence, most of the light leaves the eye through the pupil. Given that many small fishes feature at least some of the required traits, ONT eyeshine may be common among a wide range of small-sized teleost species. The exact impact of ONT eyeshine on the affected species’ visual ecology remains to be investigated.

## Methods

### Summary of experimental procedures

ONT eyeshine was discovered while observing small benthic fish in the Red Sea and Mediterranean in 2007–2012 and subsequently confirmed in the laboratory. The fishes used in this study were either wild-caught in cooperation with two Mediterranean marine research stations (*T. delaisi*, *T. melanurus*, and *P. zvonimiri*), or originated from commercial fish breeders (*A. ocellaris*). We carried out spectrophotometric measurements of the ONT eyeshine on five *T. delaisi*, two *T. melanurus*, five *P. zvonimiri*, and five *A. ocellaris.* An additional eight individuals of *T. delaisi* were used to demonstrate the ONT eyeshine’s appearance and causes, test the contribution of components of the visual pathway, and to measure the angular extension of the emitted beam of light. To induce ONT eyeshine, we illuminated a fish’s head either with a LCD projector, or a cold light source and fibre optics. Still and video footage was taken with a Nikon D4 (Nikon Corporation, Tokyo 100-8331, Japan) and quantitative measurements were made with a PR-740 spectroradiometer (Photo Research Inc., North Syracuse, NY 13212-3349, USA).

Another ten *T. delaisi*, four *T. melanurus*, one *P. zvonimiri*, and nine *A. ocellaris* were used for comparative anatomy. To obtain diverse and complementary data at different levels of detail, we split the available samples among three procedures: MRI (two *T. delaisi*, one *T. melanurus*, three *A. ocellaris*), paraffin-embedded thick histological sectioning (four *T. delaisi*, two *T. melanurus*, one *P. zvonimiri*, three *A. ocellaris*), and resin-embedded semi-thin sectioning (four *T. delaisi*, one *T. melanurus*, three *A. ocellaris*). The single *P. zvonimiri* was assigned to paraffin-embedded sectioning because this method was expected to produce more data than the other two approaches. Because data from the different approaches could usually not be combined, resulting in low independent sample sizes, analyses are restricted to descriptive statistics.

### Ecology of the investigated species

Tripterygiidae occur worldwide and generally inhabit hard substrates in the shallow littoral zone from tropical to temperate regions [44]. The genus *Tripterygion* occurs in the East Atlantic and Mediterranean, and underwent a recent radiation leading to four recognised and possibly several cryptic species [45–47]. Our focal species, *Tripterygion delaisi* Cadenat and Blache 1970, can be found across the genus’ entire distribution range, while*T. melanurus* is endemic to the Mediterranean [47]. Both species co-locate, prefer rocky substrates and prey on benthic invertebrates [48]. What distinguishes them is that they occupy different depth and light niches, which may have led to their divergence [49]. *T. delaisi* occurs between about 3 and 50 m [50], and is most abundant at depths of 6 to 12 m [44]. It shuttles between shaded microhabitats under rocks and overhangs, and exposed, sunlit surfaces [49] (Pers. obs.). *T. delaisi*’s visual system features a fovea, a cone-dominated, regular, square-mosaic pattern, is trichromatic, and restricted to visible light, since its ocular media do not transmit ultra violet light (unpublished data). *T. melanurus* can be found at depths of about 1 to 12 m, mostly between 2 and 8 m, where it inhabits only dimly and indirectly lit microhabitats, such as caves, crevices and the underside of ledges, often in association with sponges [49, 51, 52]. Its visual system has not been investigated, but may be expected to be similar to *T. delaisi*’s.

Both species live in small groups of two to six individuals from different age classes and both sexes in a loosely defined “home area” of just a few m^2^ (personal observation). Males establish and vigorously defend territories during the breeding season [44, 53, 54]. *T. delaisi* is highly cryptically coloured [49], except for breeding males, which feature a black head and a bright yellow body. Although *T. melanurus* is mostly bright red throughout the year, with a grey-to-black head, it also appears cryptic in shady crevices at depth. Most of the time, both species sit still and only move their eyes to scan the environment. They never swim continuously, but rather move in dashes and freeze again in their new position. Even when a potential predator approaches, they stay put at first, and wait for the threat to pass. Only if approached too closely or quickly, they dash away in one to several short darts, just to freeze again (Pers. obs.). *T. delaisi* is easy to catch and keep for laboratory observations and measurements.

Blenniidae and Tripterygiidae both belong to the Order Blenniiformes, although their specific relationship is not yet confirmed [55, 56]. Members of both families often form guilds in the littoral zone of the Mediterranean, which have been studied extensively, e.g., concerning depth distribution [52, 53, 57–59], trophic interactions [48, 60–63], and habitat characteristics [64]. Given these similarities, we wanted to see whether blennies, represented by *Parablennius zvonimiri*, have similar morphological features in relation to the ONT eyeshine. *P. zvonimiri* is endemic to the Mediterranean and the Black Sea, where it inhabits mostly the lower intertidal and upper subtidal zones down to about 6 m, but is most abundant between depths of 0.5 and 1.5 m [59, 65, 66]. It feeds predominantly on periphyton [65], but opportunistically also on small invertebrates (pers. obs.), and prefers vertical rock walls as a substrate, which is correlated to the availability of the piddock holes it commonly occupies [65, 67].

*Amphiprion ocellaris*, the false clown anemonefish, belongs to the family Pomacentridae and is only distantly related to the Tripterygiidae and Blenniidae families [55], all being within the order Perciformes. Anemonefishes live in hierarchically structured groups of 2–8 individuals in symbiosis with sea anemones [68]. The symbiosis with their hosts [69–71], as well as the social interactions among individuals in a group [72–74], have been studied extensively. Anemonefishes are protandrous hermaphrodites, benthic spawners with male egg guarding [75, 76], and feed mostly on zooplankton [76, 77] with a few exceptions [78]. We chose the black-and-white morph of *A. ocellaris* to assess the effect of pigmentation on the ONT eyeshine, mainly because it had the darkest pigmentation of all considered fishes in the size range of our study species.

### Origin of specimens

All triplefins (23 *Tripterygion delaisi* and 6 *Tripterygion melanurus*) and blennies (6 *Parablennius zvonimiri*) were wild-caught at either the *Centro Marino Elba* research station (Loc. Fetovaia 72, I-57034 Campo nell’Elba, Italy) in June 2013, or at STARESO research station (Pointe Revellata, BP33 20260 Calvi, Corsica, France) in June 2010, 2011, and 2016. Fish were kept in the aquarium facilities of the *Animal Evolutionary Ecology* group at the University of Tübingen until euthanized.

The clownfish (*Amphiprion ocellaris,* black-and-white morphs) originated from fish breeders. The nine individuals used for comparative anatomy were acquired on 14 August 2013 from *Oceanreefs marine aquariums* (51/7 Buckingham Drive, Wangara WA 6056, Australia) and housed temporarily in the aquaria facility of the *Neuroecology* group at The University of Western Australia until euthanized on 16 August 2013. The five individuals used for spectrophotometric measurements of their ONT eyeshine were acquired on 6 September 2016 from *Riffwelt* (Thomas-Walch-Str. 45a, A-6460 Imst, Austria). They were sacrificed and had their ONT eyeshine measured upon arrival.

### Spectrometry and emission angles of the ONT eyeshine

We conducted quantitative, spectrophotometric measurements of the ONT eyeshine for five *T. delaisi*, two *T. melanurus*, five *P. zvonimiri*, and five *A. ocellaris*. The low number of *T. melanurus* is the result from both difficulties to catch them in the wild and the death of several successfully caught individuals before we could measure them. All fish were euthanized in seawater containing a lethal dose of 500 mg/l tricaine methanesulfonate (MS-222), re-adjusted to its original pH with sodium hydroxide, immediately before taking measurements. After their opercular movement had ceased, the fish were transferred to an acrylic glass cylinder filled with more of the MS-222 solution to ensure euthanasia. Fish were fixed with pins on a piece of rubber foam. Piercing the fishes was avoided to prevent bleeding. A piece of foamed-PTFE diffuse white reflectance standard with 45°-tilted surface was pinned to the rubber foam bed next to the fish. See Additional file 6 for an overview of the set-up.

The tank was then placed on a platform that allowed vertical, rotational, and translational movements. A PR-740 SpectraScan® spectroradiometer (Photoresearch, Chatsworth CA 91311, USA), attached to a tripod, was positioned above the platform with the tank. Measurements were taken through an endoscope (No. 86190 CF) attached via a C-Mount adapter (No. 80591 C, Karl Storz GmbH & Co. KG, Industrial Group, 78532 Tuttlingen, Germany). All illumination was provided by a KL2500 LCD cold light source (Schott AG, 55127 Mainz, Germany), set to light intensity 5E and using the inbuilt cyan filter, via a 15 mm Ø optic cable (No. 250102, Schott AG). The spectroradiometer and light source remained stationary while the fishes’ eyes and the white standard were brought into position by the sole movement of the tank and platform. Nine measurements, three of each type, were taken for each eye, always in the same order: the white standard under the same illumination conditions as the subsequent measurements, serving as a proxy for the incident light intensity, then the optic disc exhibiting ONT eyeshine, and finally the surrounding retina and its potential PET eyeshine. Photographs of each fish’s optic discs were taken between measurements and directly through the spectroradiometer’s eyepiece with a Nexus 5 mobile phone camera. Data were collected with SpectraWin® (version 2.3.7, Photo Research Inc., North Syracuse, NY 13212-3349, USA), and processed and analyzed in JMP® (version 11.1.1, SAS Institute Inc., Cary, NC 27513-2414, USA).

Angular measurements were carried out in 2011 (before the main study) using six specimens from Corsica (Stareso) that had been collected in 2010 and 2011. For the emission angle measurements, the fish were placed in a small water tank and illuminated from above. The fish’s eye was then observed from a few meter distance through a monocular telescope attached to a mobile tripod. While the set-up was moved in an arch around the tank, the points where ONT eyeshine became visible and vanished again were noted. Measuring the lengths of all sides of the triangle between these two points and the fish’s eye, correcting for refraction at the glass/air interface, and applying the law of cosines, yielded the horizontal angular width of the ONT eyeshine. This procedure was repeated three times for each eye of six fish and the data then averaged per fish. The vertical angle was measured analogously by moving the tripod up and down instead of sideways for both eyes in five out of the original six fish.

### Video documentation and ONT eyeshine generation pathway

We used one *T. delaisi* to photo- and video-document its ONT eyeshine under controlled conditions. The fish was transferred to a cylindrical, acrylic glass observation tank that rested on a turntable. This allowed us to adjust the horizontal angle. The fish was illuminated by two KL2500 LCD cold light sources (Schott AG, 55127 Mainz, Germany). One provided diffuse background illumination of the whole animal via a 15 mm Ø optic cable (No. 250102, Schott AG), and the other induction illumination for ONT eyeshine via a 3 mm Ø optic cable (No. 155101, Schott AG) that ended approximately 5 mm above the animal’s head and illuminated only a 4 mm wide area above and behind the eyes. This allowed us to change the colours of the background and induction independently, and thus highlight the origin of the light contributing to ONT eyeshine. All photographs and videos were taken with a Nikon D4 (Nikon Corporation, Tokyo 100-8331, Japan).

After all footage from the live fish had been taken, the animal was sacrificed in a MS-222 bath as described previously. The fish was kept in physiological saline for marine teleosts [79] and used to document light transmission under a reversed light path. To that end we shone a narrow beam of light from a 1 mm Ø optic cable (FC UV1000-2, Avantes BV, NL-7333 NS Apeldoorn, Netherlands) through the pupil of one eye onto the optic disc and recorded which areas of the skull lit up most. As a control, we also illuminated other parts of the fish’s head to document light transmission through normal scattering. To test the different brain areas’ contribution to ONT eyeshine, we removed the skin and skullcap (posterior frontals and parietals) between and directly behind the eyes. Then a single glass fibre was positioned directly above the area of interest, limiting direct illumination only to the tested structure. Brain parts tested in this way included cerebellum, optic tecta, left and right sides of the telencephalon, and left and right ONs. The resulting ONT eyeshine was recorded photographically with the Nikon D4, using the same, manually fixed settings for all images. The ONT eyeshine’s brightness was later assessed by measuring the average grey values of the pupil area in the images using ImageJ (version 1.46q; https://imagej.nih.gov/ij/index.html).

In another *T. delaisi*, we mapped which area of the nape and interorbital region of the skull shows the highest efficiency in generating ONT eyeshine. For this purpose, an LCD projector was positioned 2 m above a small tank with a living fish in a dark room. Due to their cryptobenthic nature, *T. delaisi* individuals usually sit inactive in the dark with no signs of increased activity, breathing, or stress. A laptop was used to produce a single white pixel that could be moved around on the otherwise black screen using the touchpad of an IBM laptop computer. By focusing the pixel on the head of the fish, it was possible to move it across the fish head and scan the whole fish in approximately 1 mm^2^ sized squares, covering the whole head from the tip to the first dorsal fin. At each position, the strength of the ONT eyeshine was visually assessed in three steps: 0 (no ONT eyeshine), 1 (weak ONT eyeshine) and 2 (bright ONT eyeshine). We assigned these values to the corresponding grid coordinates on a photograph of the fish. The data of the left eye was mirrored and combined with that of the right eye to produce the excitation map in Fig. 4a.

### Histology I: semi-thin, resin-embedded sections

Semi-thin histological sections were produced from four *T. delaisi* (Td5-8), one *T. melanurus* (Tm3), and three *A. ocellaris* (Ao1-3). Euthanasia was carried out in a solution of seawater and 500 mg/l MS-222, as previously. Exposure lasted at least 2 min or until the following conditions were met: ceased opercular movement and complete loss of buoyancy control for 1 min. Thereafter, the spinal cord was severed to ensure euthanasia. The total length, head width, and head height were measured, then the fish were decapitated, and their heads trimmed to the respective regions of interest. These samples were then fixed and stored according to the protocols described below. Subsequent histological processing and MRI scans were carried out at UWA (Perth) and UQ (Brisbane). *Tripterygion* and *Parablennius* samples were therefore imported to Australia according to DAFF regulations (permit no. IP13006051). The lower jaw was removed and the remaining head cropped, leaving a region from the rostral edge of the eyes to the caudal edge of the cranium. The sample was subsequently immersion fixed in a modified Karnovsky’s solution containing 2% paraformaldehyde (PFA), 1.5% glutaraldehyde (GA), and 1% dimethyl sulfoxide (DMSO) in 0.155 M, pH = 7.3, Sorensen’s phosphate buffer (SPB). Then, samples were washed once with a glycine solution to block residual aldehyde groups (1% DMSO and 1% glycine in 0.155 M, pH = 7.3, SPB). Finally, samples were washed two more times and eventually stored in 0.155 M, pH = 7.3, SPB containing 1% DMSO and 0.1% sodium azide, which prevents bacterial and fungal growth. Each washing step lasted 24 h.

Sample preparation continued without decalcification in a Lynx EL automated tissue processor. To fit the baskets, all heads were split medially and further trimmed to the caudal region of the eye and the ON. The tissue processor dehydrated the samples through an ascending ethanol series and infiltrated them with epoxy resin, using propylene oxide as an intermediary solvent overnight. Infiltrated samples were embedded in resin blocks and polymerised at 60 °C for 72 h. The resin blocks were sectioned with a LKB Bromma Ultratome NOVA. Glass knives were used to skim through the blocks and position was controlled every 50 μm. Once within the target area, consisting of optic disk, surrounding retina and ON, three to four 1-μm-sections were cut, using a DiATOM ultra diamond knife (45° angle), before skimming another 50 μm with a glass knife. Successful target sections were transferred to water droplets on a positively charged glass slide and put on a hotplate (90 °C) to expand and dry. Sections were stained with Toluidine blue after the resin was removed with alcoholic sodium hydroxide, and mounted using Entellan® (Merck Millipore, 64293 Darmstadt, Germany).

### Histology II: thick, paraffin-embedded sections

Four *T. delaisi* (Td1-4), two *T. melanurus* (Tm1-2), one *P. zvonimiri* (Pz1)*,* and three *A. ocellaris* (Ao4-6) were prepared for paraffin-embedded thick sections as follows:

Euthanasia and sample trimming were carried out as described above. The samples were then immersion-fixed in 0.155 M SPB at pH = 7.3, containing 4% PFA and 1% DMSO. The fixed samples were washed three to four times, each lasting 24 h, with 0.155 M, pH = 7.3, SPB containing 1% DMSO and 0.1% sodium azide. The same solution also served sample storage. To allow cutting with steel blades, the tissue was furthermore decalcified in citrate (7.5%) buffered formic acid (15%) for 48 h. Larger specimens were split medially to facilitate processing.

Samples were dehydrated in an ascending ethanol series, cleared in xylene and infiltrated with paraffin in a Shandon Citadel^TM^ 2000 tissue-processing carousel. When embedding them in paraffin blocks, samples were oriented such that whole-head specimens were cut in the transverse plane, while one half of split-head specimens was cut coronally, the other cut sagittally. Blocks were sectioned on an AO® rotary microtome (model 820) at 10 μm section thickness using a steel blade. Sections (ribbons of 5–10) were floated in a water bath (43 °C, degassed and deionised water, 0.01% gelatine) to flatten and expand, and then transferred to standard glass slides and dried overnight at 60 °C in a heating cabinet.

Samples were then subjected to a modified phosphotungstic acid haematoxylin (PTAH) staining protocol [80], which stains several tissue types simultaneously and differently, e.g., muscles dark purple, connective tissue reddish-brown, and neuroglia (including myelin sheaths) deep-blue. We proceeded as follows: The sections were first dewaxed in xylene and rehydrated through a descending ethanol series. Then they were postfixed in 3% potassium dichromate solution for 20 min and rinsed with water afterwards. Next, the sections were immersed in 0.25% acidified potassium permanganate solution for 1 min, rinsed in water, and then bleached in 2% oxalic acid until clear and rinsed in water. Finally, sections were stained overnight in the PTAH solution. This solution needs to be prepared in advance as follows: For a batch of 500 ml, individually dissolve 0.5 g haematoxylin, 10 g phosphotungstic acid and 0.0625 g potassium permanganate in 100 ml, 375 ml and 25 ml distilled water, respectively; mix the component solutions and allow ripening at least overnight, best for a week; the solution’s shelf life is several weeks. After staining, the sections were dehydrated through an ascending ethanol series and cleared once more in xylene, before mounting them with Entellan® (Merck Millipore, 64293 Darmstadt, Germany).

### Magnetic resonance imaging

MRI was performed on two *T. delaisi* (Td9-10), one *T. melanurus* (Tm5), and three *A. ocellaris* (Ao7-9). After euthanizing the fishes as described previously, their heads were trimmed to upper jaw, eyes, and cranium. Samples were immersion-fixed for 24 h in phosphate buffered saline (PBS) containing 4% PFA and 0.25% of 1 M Gadovist® (Bayer AG, 51373 Leverkusen, Germany), and washed three to four times, 24 h each, in PBS with 0.1% sodium azide and 0.25% 1 M Gadovist® at pH = 7.3. Gadovist® is a gadolinium-based contrast agent for MRI scans. Limited availability of the MRI scanner led to several weeks delay between sample fixation and scanning. Specimens were stored in the final washing step solution at 4 °C for the intervening time.

All scans were acquired overnight on a 16.4 T Ultrashield™ Plus 700 WB Avance NMR spectrometer running ParaVision® 5.1 software (both from Bruker BioSpin GmbH, 76287 Rheinstetten, Germany) at the Centre for Advanced Imaging, University of Queensland, Brisbane, Australia. For most fish (Ao7-9, Td9) individual scans were obtained and processed. Tm5 and Td10, however, were imaged and processed together, resulting in a few divergent parameters compared to the other specimens. Acquisition parameters of the T_1_-weighted 3D FLASH scans were as follows: Repetition time 40 ms, echo time 4.8 ms (Tm5/Td10: 15.2 ms), number of excitations 9 (Tm5/Td10: 8), flip angle 45°, field of view 7 × 7 × 7.7 mm (Tm5/Td10: 7 × 7 × 20.5 mm), image matrix 464/464/512 (Tm5/Td10: 464/464/1216), isotropic resolution 15 μm (Tm5/Td10 slightly anisotropic 15 × 15 × 17 μm).

We used the MRI data to create digital 3D segmentations of the structures we suspected to be involved in the ONT eyeshine by being part of the light path. To that end we used the segmentation programme ITK-SNAP, version 2.4.0 from 21 November 2012, developed by Yushkevich et al. [81]. ITK-SNAP features a user-guided automatic segmentation, which we used for a draft segmentation that we then corrected and refined manually, where necessary.

### Digital image processing, measurements and statistical analysis

Both paraffin-embedded histological sections and MRI scans were used to obtain morphological data for between and within-individual comparisons as follows: All histological thick sections from the region of interest of each respective fish sample were digitalised using a Leica DM5000 B microscope equipped with a Leica DFC320 camera (Leica Microsystems, 35578 Wetzlar, Germany). The resulting TIFF images were resized and centred in Adobe® Photoshop® CS4 11.0.2 for easier processing. Individual images were then aligned and stacked using the Elastic Alignment and Montage plugin [82] in ImageJ 1.48t (Fiji distribution package). MRI images for non-volumetric measurements were obtained by exporting individual images from ITK-SNAP via the built-in snapshot function.

All linear, angular and areal measurements from digital images were taken in ImageJ 1.48t (Fiji distribution package). Measurements along the ON were taken in 40–60 μm steps between optic disc and optic chiasm in both the transverse and sagittal planes. This resulted in 10 to 21 sampled sections per fish, depending on the length of the ON. In all such sections, the length and angle of each individual layer, as well as the overall cross-sectional area (CSA) of the ON were taken. These raw measurements were then averaged per sampled section, fish, and genus, depending on what was to be compared. Absolute measurements were corrected for size, i.e., divided by the estimated body volume or the mean head diameter, where possible and applicable. These relative values were corrected for allometric relationships with body size, where the appropriate allometric factor was available.

ON layer angles and torsion were derived from transverse sections and transverse projections of aligned stacks with a different section plane. With angle of an ON layer, we mean its tilt in relation to the horizontal. The change of average layer angle along the nerve in °/mm is referred to as ON torsion. The layers in the ON represent bidirectional axes rather than unidirectional vectors, i.e., both angles α = 180° and α’ = 0° correspond to the same horizontal layer. Hence, we considered any angle α = x° equivalent to α’ = (x ± 180)° and transformed some of the raw angles accordingly such that the difference between smallest and largest individual angle in a sample, i.e., the circular range, was minimised. This procedure not only facilitated the calculation of mean angle and circular standard deviation, but also made the resulting means more reliable.

The following equations were used for all angular calculations:

Mean angle calculation:

1 $$ \overline{a}= \arctan \left(\frac{ \sin \overline{a}}{ \cos \overline{a}}\right) $$

with

2 $$ \begin{array}{ccc}\hfill \sin \overline{a}={\displaystyle \sum_{i=1}^n{ \sin}_{a_i}{l}_{r_i}}\hfill & \hfill \mathrm{and}\hfill & \hfill \cos \overline{a}={\displaystyle \sum_{i=1}^n{ \cos}_{a_i}{l}_{r_i}}\hfill \end{array} $$

and

3 $$ {l}_{r_i}=\frac{l_i}{{\displaystyle {\sum}_{i=1}^n{l}_i}} $$

*ā*: Mean sample angle
*l*_*r*_: Relative layer length

Circular standard deviation calculation:

4 $$ v=\sqrt[2]{-2 \ln \left(\overline{R}\right)} $$

with

5 $$ \overline{R}=\sqrt[2]{{\left( \sin \overline{a}\right)}^2+{\left( \cos \overline{a}\right)}^2} $$

*V*: Sample circular standard deviation
$$ \overline{R} $$
Sample mean resultant length (mean angle vector length).

Modified from [75] and [76].

The mean angles were calculated according to Eq. 1, weighting each individual angle by the relative length of the respective layer (Eqs. 2–3), and the circular standard deviation according to Eqs. 4-5. To quantify the torsion of the ON, we plotted mean angles of sampled transverse sections against distance from optic disc and fitted a linear function to the data. Assuming a continuous and constant torsion, we equivalently transformed mean angle values to reduce large steps between data points, where necessary. Where an estimated parameter of an individual linear fit was not significantly different from zero, we set its value to zero. We then averaged these parameters to obtain mean linear fits of the ON torsion per genus.

All previous calculations and statistical analyses of the resulting data were carried out in JMP® (version 11.1.1, SAS Institute Inc., Cary, NC 27513-2414, USA). Although we used a total number of 14 *A. ocellaris*, six *P.* zvonimiri, 21 *T. delaisi*, and six *T. melanurus* in this study, we could take some measurements only from a subset of these fish. Therefore, sample sizes may differ and are given separately for each type of measurements. Statistical tests were only applied when sample sizes allowed them. For most of the data, however, only descriptive statistics could be calculated and are given as mean ± standard deviation, unless stated otherwise.

### Visual model of potential signalling function

To investigate the potential role of ONT eyeshine in intraspecific signalling, we assessed the effect of the ONT eyeshine on the achromatic and chromatic contrast between the triplefin pupil with and without the eyeshine, as it would be perceived by a conspecific. Our calculations are based on the receptor-noise colour discrimination model described by Vorobyev and Osorio [83] and implemented in the R package pavo [84]. In these neural noise models, we included the photoreceptor sensitivity curves of *T. delaisi* (λmax: single cone – 468 nm, double cone – 516 and 530 nm, treated as trichromat, [85]), which were generated using the visual template equation developed by [86], the light transmission properties of the ocular media of *T. delaisi*, a photoreceptor density ratio of 1:4:4 (unpublished data) and a Weber fraction of 0.05 for the most abundant photoreceptor type. We further assumed that downwelling irradiance was the main contributor to the ONT eyeshine, and used the pigment-epithelium transmitted eyeshine as basis for comparison. We used the transmission values from the measurement with the overall brightest ONT eyeshine for each of the five triplefins, and the ambient irradiance values from the field. The calculation of the achromatic contrasts was based on the sensitivity curves of the double cone photopigment. Values of ΔS and ΔL greater than one just-noticeable-difference (JND) indicate that the contrast between two signals would be discriminable while values of less than 1 JND would indicate that the contrast between the two signals would not be discernible [83].

### Data sources for visual model

#### Downwelling irradiance

Downwelling irradiance was measured in 0.5 m depth intervals on 15 June 2011 (around noon, sunny weather) between 0 and 10 m while scuba diving near Stareso field station, Calvi, Corsica. We used a calibrated PR 670 PhotoResearch radiospectrometer in a custom underwater housing (UK-Germany) and measured the radiance of (1) an exposed white reflectance standard and (2) a shaded white reflectance standard. Both standards had their surface positioned vertically and facing South, to simulate the combined direct and scattered light reaching and being radiated off the side of a fish. The shaded standard had a hood made of black anodised aluminium foil that blocked out direct light. Radiance spectra in Watts/sr/m^2^/nm were transformed into photon irradiance (photons/s/m^2^/nm) following [87], and used to calculate attenuation coefficients as explained in [88]. With these attenuation values we recalculated the expected irradiance at the required depth, using the irradiance value measured just below the water surface as the total amount of incoming light.

#### Photoreceptor sensitivities

The photoreceptor sensitivities of *T. delaisi* were supplied by Connor M. Champ and Shelby Temple (unpublished data), who used microspectrometry (MSP) on eyes from 7 individual fish in the laboratory of Christian Donner at the University of Helsinki, following the general method proposed by Govardovskii et al. [86].

#### Ocular media properties

NKM dissected the eyes of 8 individual *T. delaisi* in Stareso, Calvi, Corsica, in June 2012. The dermal (external) and scleral (internal) corneas, as well as the lens were individually placed in a petri dish with isotonic, marine Ringer buffer. The dish was placed under a stereomicroscope equipped with a PR 670 PhotoResearch Radiospectrometer, and illuminated from below with a KL 2500 (Schott) cold light source. The radiance of the light source was repeatedly and alternatingly measured through the Petri dish and buffer alone (L_0_), and through dish, buffer, and ocular tissue sample (L_OT_) in 1 nm steps. Wavelength-specific transmittance was calculated as T(λ) = L_OT_(λ)/L_0_(λ). For the model, average transmittance was used.

## Additional files

Additional file 5: Table with modelled effects of ONT eyeshine on perceived contrast of the eye. Comprehensive modelling data that show the contrast changes of pupil and iris caused by exhibiting ONT eyeshine, as perceived by another *T. delaisi,* to assess the potential role of ONT eyeshine in intraspecific communication and signalling. (PDF 59 kb)
Additional file 6: Figure showing overview of spectrophotometry set-up. Set-up used for the spectrophotometric measurements of the ONT eyeshine in the studied species. 1) spectroradiometer; 2) endoscope, attached via a C-Mount adapter; 3) tripod; 4) cold light source, using the inbuilt cyan filter; 5) optic cable; 6) platform that allowed for controlled vertical movements; 7) cylindrical acrylic glass tank that could be rotated and displaced horizontally on the platform; 8) rubber foam; 9) diffuse white reflectance standard made of foamed PTFE; 10) fish, euthanized and immobilised with pins; 11) laptop running SpectraWin®, version 2.3.7, for data collection. (PNG 282 kb)
Additional file 7: Table with comprehensive, absolute anatomical data, the summary of which appears in Table 1. (PDF 75 kb)
Additional file 8: Table with comprehensive, relative anatomical data, the summary of which appears in Table 1. (PDF 102 kb)
Additional file 9: Table with full optic nerve torsion data and parameters, on which the summary in Table 2 was based. (PDF 61 kb)

## Abbreviations

CSA: Cross-sectioned area
CTR: Choroidal-tapetum-reflected
ICR: Iridescent-cornea-reflected
MRI: Magnetic resonance imaging
ON: Optic nerve
ONT: Optic-nerve-transmitted
PET: Pigment-epithelium-transmitted
PTAH: Phosphotungstic acid haematoxylin
RPE: Retinal pigment epithelium
RTR: Retinal-tapetum-reflected
SAR: Stratum-argenteum-reflected
