# Supplementary material for: Optic-nerve-transmitted eyeshine, a new type of light emission from fish eyes
Source: Front Zool. 2017 Feb 27;14:14. doi: 10.1186/s12983-017-0198-9 (PMC5327540; doi:10.1186/s12983-017-0198-9)
Supplement: Additional file 5: — Table with modelled effects of ONT eyeshine on perceived contrast of the eye. Comprehensive modelling data that show the contrast changes of pupil and iris caused by exhibiting ONT eyeshine, as perceived by another T. delaisi, to assess the potential role of ONT eyeshine in intraspecific communication and signalling. (PDF 59 kb) [file 12983_2017_198_MOESM5_ESM.pdf]

Add. File 09: Chromatic and achromatic contrast between the pupil with and without the optic nerve transmitted eyeshine, as perceived by *T. delaisi*, to assess the potential for intraspecific communication. The model assumes a trichromatic visual system for *T. delaisi* based on micro-spectrophotometric data (unpublished). Chromatic difference values and achromatic difference values are expressed in just-noticeable-differences. See Materials and Methods for more details on model parameters and explanation of values presented.

**5 m depth**		**20 m depth**		
**Chromatic**	**Achromatic**	**Chromatic**	**Achromatic**	
0.50	7.59	0.41	7.86	**individual *T. delaisi***
0.44	6.56	0.34	6.81	
0.41	3.90	0.34	4.09	
0.19	2.82	0.15	2.94	
0.20	3.39	0.16	3.49	
0.35	4.85	0.97	5.04	**mean**
