# Supplementary figures and images for: Optic-nerve-transmitted eyeshine, a new type of light emission from fish eyes
Source: Front Zool. 2017 Feb 27;14:14. doi: 10.1186/s12983-017-0198-9 (PMC5327540; doi:10.1186/s12983-017-0198-9)

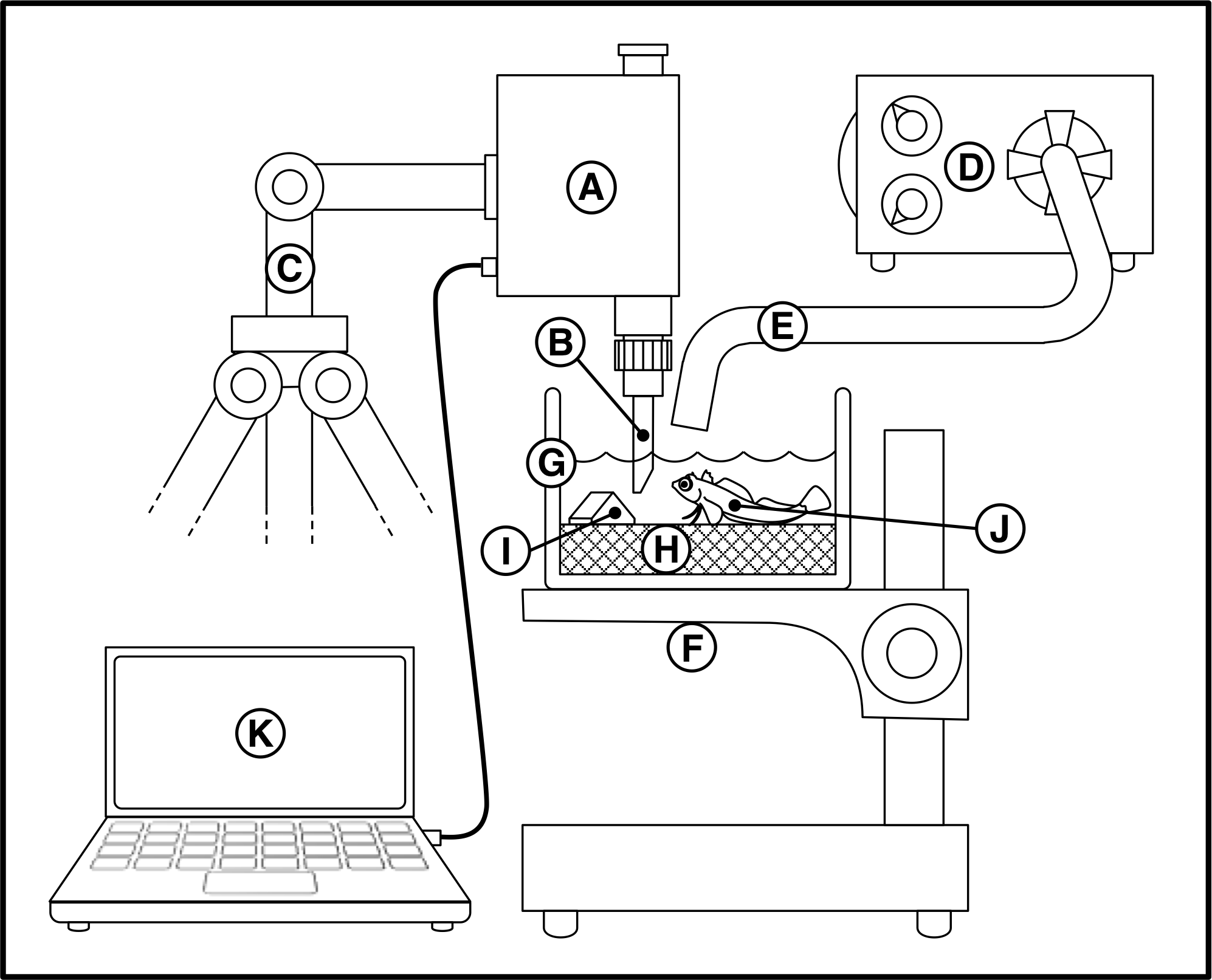

Supplement: Additional file 6: — Figure showing overview of spectrophotometry set-up. Set-up used for the spectrophotometric measurements of the ONT eyeshine in the studied species. 1) spectroradiometer; 2) endoscope, attached via a C-Mount adapter; 3) tripod; 4) cold light source, using the inbuilt cyan filter; 5) optic cable; 6) platform that allowed for controlled vertical movements; 7) cylindrical acrylic glass tank that could be rotated and displaced horizontally on the platform; 8) rubber foam; 9) diffuse white reflectance standard made of foamed PTFE; 10) fish, euthanized and immobilised with pins; 11) laptop running SpectraWin®, version 2.3.7, for data collection. (PNG 282 kb) [file 12983_2017_198_MOESM6_ESM.png]
