# Supplementary material for: Optic-nerve-transmitted eyeshine, a new type of light emission from fish eyes
Source: Front Zool. 2017 Feb 27;14:14. doi: 10.1186/s12983-017-0198-9 (PMC5327540; doi:10.1186/s12983-017-0198-9)
Supplement: Additional file 7: — Table with comprehensive, absolute anatomical data, the summary of which appears in Table 1. (PDF 75 kb) [file 12983_2017_198_MOESM7_ESM.pdf]

Add. File 06: Overview of individual, absolute anatomical data and summary per species.

fish	section type	TL (mm)	MHD (mm)	EBV (mm³)	Skull (bone) thickness ± SD_{intra} (µm)	Dermis thickness ± SD_{intra} (µm)	ON depth ± SD_{intra} (mm)	ON layers (#)	ON layer thickness ± SD_{intra} (µm)	Mean ON CSA ± SD_{intra} (mm²)	Comments
Td1	10 µm	62	7.5	913	-	-	-	7	60.0 ± 8.1	0.170 ± 0.046	
Td2	10 µm	52	6.8	629	-	-	-	8	64.9 ± 14.9	0.144 ± 0.055	
Td3	10 µm	57	7.1	752	-	-	-	-	-	-	section series compromised
Td4	10 µm	50	6.6	570	53.3 ± 20.3	89.5 ± 30.0	1.61 ± 0.18	8.5	39.7 ± 7.1	0.092 ± 0.024	
Td5	1 µm	47	6.6	536	-	-	-	-	-	-	* Only used for qualitative intra-
Td6	1 µm	50	6.6	570	-	-	-	-	-	-	ocular ON and retina structure.
Td7	1 µm	56	7	718	-	-	-	-	-	-	Since cropped to ROI, measure-
Td8	1 µm	50	6.5	553	-	-	-	-	-	-	ments would be incomplete.
Td9	MRI	58	7.5	854	61.3 ± 15.6	89.6 ± 43.7	1.57 ± 0.37	-	-	-	
Td10	MRI	54	7.25	743	47.5 ± 9.9	103.4 ± 24.8	1.53 ± 0.25	-	-	-	
T. delaisi **Mean ± SD**		**53.6 ± 4.6**	**6.9 ± 0.4**	**684 ± 133**	**54.0 ± 6.9**	**94.2 ± 8.0**	**1.57 ± 0.04**	**7.8 ± 0.8**	**54.9 ± 13.4**	**0.135 ± 0.040**	
Tm1	10 µm	45	5.05	300	-	-	-	6	47.9 ± 8.4	0.089 ± 0.021	
Tm2	10 µm	34	4.3	165	33 ± 8.9	30.9 ± 19.8	1.05 ± 0.07	7	32.2 ± 3.8	0.040 ± 0.011	
Tm3	1 µm	39	4.55	211	-	-	-	-	-	-	see *
Tm4	MRI	44	5.55	355	43.6 ± 11.3	39.6 ± 13.7	1.12 ± 0.25	-	-	-	
T. melanurus **Mean ± SD**		**40.5 ± 5.1**	**4.9 ± 0.6**	**258 ± 86**	**38.3 ± 7.5**	**35.3 ± 6.2**	**1.09 ± 0.05**	**6.5 ± 0.7**	**40.1 ± 11.1**	**0.065 ± 0.035**	
P. zvonimiri	10 µm	42	5.55	339	84.2 ± 54.7	260.6 ± 160.4	1.38 ± 0.21	4.5	63.5 ± 8.4	0.124 ± 0.037	
Ao1	1 µm	56	10.7	1679	-	-	-	-	-	-	
Ao2	1 µm	47	8.6	910	-	-	-	-	-	-	see *
Ao3	1 µm	47	8.5	889	-	-	-	-	-	-	
Ao4	10 µm	57	10.2	1553	-	-	-	10	46.7 ± 4.7	0.089 ± 0.019	
Ao5	10 µm	56	10.65	1663	-	-	-	11	43.3 ± 9.0	0.096 ± 0.012	
Ao6	10 µm	50	9.3	1132	232.6 ± 92.0	207.4 ± 118.1	1.84 ± 0.16	9.5	38.1 ± 8.0	0.104 ± 0.013	
Ao7	MRI	52	10.2	1416	227.5 ± 64.7	224.6 ± 104.6	2.09 ± 0.21	-	-	-	
Ao8	MRI	49	9.05	1051	248.9 ± 68.3	281.2 ± 127.2	2.12 ± 0.18	-	-	-	
Ao9	MRI	62	12.35	2476	313.1 ± 103.3	386.8 ± 174.2	2.58 ± 0.12	-	-	-	
A. ocellaris **Mean ± SD**		**52.9 ± 5.2**	**10.0 ± 1.2**	**1419 ± 503**	**255.5 ± 39.5**	**275.0 ± 80.9**	**2.16 ± 0.31**	**10.2 ± 0.8**	**42.7 ± 4.3**	**0.096 ± 0.007**	

CSA = Cross-sectional area; EBV = Estimated body volume; MHD = Mean head diameter; ON = Optic nerve; ROI = region of interest; SD = standard deviation of means per species; SD_{intra} = SD of subsamples within individual, TL = Total length
