# Supplementary material for: Optic-nerve-transmitted eyeshine, a new type of light emission from fish eyes
Source: Front Zool. 2017 Feb 27;14:14. doi: 10.1186/s12983-017-0198-9 (PMC5327540; doi:10.1186/s12983-017-0198-9)
Supplement: Additional file 8: — Table with comprehensive, relative anatomical data, the summary of which appears in Table 1. (PDF 102 kb) [file 12983_2017_198_MOESM8_ESM.pdf]

Add. File 07: Overview of relative, size-corrected, individual data and summary per species.

fish	Skull (bone) thickness ± SD_{intra} (% of MHD)	Dermis thickness ± SD_{intra} (% of MHD)	ON depth ± SD_{intra} (% of MHD)	ON layer thickness ± SD_{intra} (% of AON Ø)	Mean ON CSA ± SD_{intra} (% of Head CSA)
Td1	-	-	-	12.9 ± 1.7	0.38 ± 0.10
Td2	-	-	-	15.2 ± 3.5	0.40 ± 0.15
Td4	0.81 ± 0.31	1.36 ± 0.45	24.4 ± 2.7	11.6 ± 2.1	0.27 ± 0.07
Td9	0.82 ± 0.21	1.19 ± 0.58	20.9 ± 4.9	-	-
Td10	0.66 ± 0.14	1.43 ± 0.34	21.1 ± 3.4	-	-
T. delaisi **Mean ± SD**	**0.76 ± 0.09**	**1.33 ± 0.12**	**22.1 ± 2.0**	**13.2 ± 2.1**	**0.35 ± 0.07**
Tm1	-	-	-	14.2 ± 2.5	0.44 ± 0.10
Tm2	0.77 ± 0.21	0.72 ± 0.46	24.4 ± 1.6	14.3 ± 1.7	0.28 ± 0.08
Tm4	0.79 ± 0.20	0.71 ± 0.25	20.2 ± 4.5	-	-
T. melanurus **Mean ± SD**	**0.78 ± 0.01**	**0.72 ± 0.004**	**22.3 ± 3.0**	**14.2 ± 0.03**	**0.36 ± 0.12**
P. zvonimiri	1.52 ± 0.99	4.70 ± 2.89	24.9 ± 3.8	16.0 ± 2.1	0.51 ± 0.15
Ao4	-	-	-	13.9 ± 1.4	0.11 ± 0.02
Ao5	-	-	-	12.4 ± 2.6	0.11 ± 0.01
Ao6	2.50 ± 0.99	2.23 ± 1.27	19.8 ± 1.7	10.5 ± 2.2	0.15 ± 0.02
Ao7	2.23 ± 0.63	2.20 ± 1.03	20.5 ± 2.1	-	-
Ao8	2.75 ± 0.75	3.11 ± 1.41	23.4 ± 2.0	-	-
Ao9	2.54 ± 0.84	3.13 ± 1.41	20.9 ± 1.0	-	-
A. ocellaris **Mean ± SD**	**2.50 ± 0.21**	**2.67 ± 0.52**	**21.1 ± 1.6**	**12.2 ± 1.7**	**0.12 ± 0.03**

AON Ø = approximated optic nerve diameter ($2 \cdot \sqrt{\frac{\text{ON CSA}}{\pi}}$); CSA = cross-section area; MHD = mean head diameter; SD = standard deviation of means per species; SD_{intra} = SD of subsamples within individual
