# Supplementary material for: Optic-nerve-transmitted eyeshine, a new type of light emission from fish eyes
Source: Front Zool. 2017 Feb 27;14:14. doi: 10.1186/s12983-017-0198-9 (PMC5327540; doi:10.1186/s12983-017-0198-9)
Supplement: Additional file 9: — Table with full optic nerve torsion data and parameters, on which the summary in Table 2 was based. (PDF 61 kb) [file 12983_2017_198_MOESM9_ESM.pdf]

Add. File 08: Optic nerve torsion linear fit parameter estimates.

fish	eye	ON length [mm]	N	R^2	Starting angle [°]	P_S	Torsion [°/mm]	P_T	Total torsion [°]
Td1	Left	0.91	17	0.883	0.5 (0)	0.9008	58.2	0.0001	52.7
	Right	0.99	15	0.934	49.2	0.0001	139.2	0.0001	137.8
Td2	Left	0.71	9	0.636	41.8	0.0001	93.8	0.0021	66.3
Td4	Left	0.99	15	0.972	12.4	0.0005	98.5	0.0001	97.5
	Right	0.99	15	0.957	17.1	0.0001	94.0	0.0001	93.1
Tm1	Left	0.74	14	0.914	19.2	0.0001	59.6	0.0001	43.8
	Right	0.51	10	0.822	10.8 (0)	0.0601	99.0	0.0003	50.4
Tm2	Left	0.68	13	0.977	18.8	0.0001	124.9	0.0001	84.8
	Right	0.85	13	0.868	8.5 (0)	0.0754	73.2	0.0001	62.1
**Tripterygion Mean ± SD**		**0.81 ± 0.16**	**5**	**0.859 ± 0.130**	**19.3 ± 9.6**		**93.4 ± 8.2**		**75.5 ± 20.5**
Pz1	Left	0.96	18	0.759	13.2 (0)	0.087	72.9	0.0001	70.1
	Right	0.96	18	0.898	25.1	0.0007	84.5	0.0001	81.3
**Parablennius Mean**		**0.96**	**1**	**0.828**	**12.5**		**78.7**		**75.7**
Ao4	Left	1.41	21	0.904	6.6 (0)	0.2069	81.7	0.0001	115.6
Ao5	Left	1.48	22	0.705	34.7	0.0087	95.1	0.0001	141.2
Ao6	Left	1.41	21	0.923	3.8 (0)	0.1876	63.1	0.0001	89.3
	Right	1.20	18	0.877	0.6 (0)	0.886	78.3	0.0001	94.1
**Amphiprion Mean ± SD**		**1.40 ± 0.09**	**3**	**0.836 ± 0.114**	**11.6 ± 20.0**		**82.5 ± 12.2**		**116.2 ± 24.8**

N indicates sampled sections per nerve or individuals for mean calculation (bold). (0) indicates parameter not significantly different from zero, hence 0 was used in mean calculation. Parameters were first averaged per individual (not shown, except *P. z.*), then per genus (bold).
